# Assessment of the quality of the healing process in experimentally induced skin lesions treated with autologous platelet concentrate associated or unassociated with allogeneic mesenchymal stem cells: preliminary results in a large animal model

**DOI:** 10.3389/fvets.2023.1219833

**Published:** 2023-07-25

**Authors:** Ilaria Iacopetti, Anna Perazzi, Marco Patruno, Barbara Contiero, Anna Carolo, Tiziana Martinello, Luca Melotti

**Affiliations:** ^1^Department of Animal Medicine, Production and Health, University of Padua, Padova, Italy; ^2^Department of Comparative Biomedicine and Food Science, University of Padua, Padova, Italy; ^3^Department of Veterinary Medicine, University of Bari, Bari, Italy

**Keywords:** skin lesion, healing process, sheep, platelet rich plasma, mesenchymal stem cells

## Abstract

Regenerative medicine for the treatment of skin lesions is an innovative and rapidly developing field that aims to promote wound healing and restore the skin to its original condition before injury. Over the years, different topical treatments have been evaluated to improve skin wound healing and, among them, mesenchymal stem cells (MSCs) and platelet-rich plasma (PRP) have shown promising results for this purpose. This study sought to evaluate the quality of the healing process in experimentally induced full-thickness skin lesions treated with PRP associated or unassociated with MSCs in a sheep second intention wound healing model. After having surgically created full-thickness wounds on the back of three sheep, the wound healing process was assessed by performing clinical evaluations, histopathological examinations, and molecular analysis. Treated wounds showed a reduction of inflammation and contraction along with an increased re-epithelialization rate and better maturation of the granulation tissue compared to untreated lesions. In particular, the combined treatment regulated the expression of collagen types I and III resulting in a proper resolution of the granulation tissue contrary to what was observed in untreated wounds; moreover, it led to a better maturation and organization of skin adnexa and collagen fibers in the repaired skin compared to untreated and PRP-treated wounds. Overall, both treatments improved the wound healing process compared to untreated wounds. Wounds treated with PRP and MSCs showed a healing progression that qualitatively resembles a *restitutio ad integrum* of the repaired skin, showing features typical of a mature healthy dermis.

## Introduction

The skin is the largest organ of the body and has the crucial role to function as a barrier from external agents. When injured, there is a loss of integrity of the skin barrier leading to a functional disorder that might be accompanied by disability or even death. Skin injury initiates mechanisms that aim to limit damage and subsequently induce repair (1, 2). Skin wound healing is a complex dynamic process that involves the interaction of multiple cell types and different soluble molecules such as growth factors, cytokines, and chemokines (3–5). It consists of four overlapping phases (hemostasis, inflammation, proliferation, and remodeling) with the aim to clean the wound bed, generate new tissue, and close the wound in order to restore the skin barrier (1, 2, 4, 5). An impairment to this mechanism can result in non-healing or chronic open wounds, but also lead to the deposition of excessive granulation tissue presented as keloids or hypertrophic scars (1, 2, 4, 6, 7). Repair of skin wounds in adults is commonly achieved with fibrosis, resulting in a scar tissue that is stiffer than healthy skin because it consists of disorganized extracellular matrix (ECM). This repair process differs from the regenerative process, as in this case the resulting tissue is almost indistinguishable from the original tissue in terms of structure and function (1). The main focus of clinical skin wound management is to promote and sustain a proper wound repair with the recovery of tissue function and a newly esthetically satisfying formed tissue (1). Regenerative skin wound therapy is a novel and rapidly developing field of biomedical research that aims to promote wound healing and to restore the damaged cells and injured skin tissue without scar formation. Since quality care is a crucial aspect of wound healing, regenerative strategies should not be considered as an alternative for certain indispensable conventional treatments; instead, they should be considered complementary (1, 6, 7). Several *in vitro* and *in vivo* studies have been conducted on regenerative strategies for wound healing in humans and different animal species. Regenerative therapies consist of different strategies, such as the application of growth factors, gene therapy, stem cell treatment, tissue engineering, and cell reprogramming (1, 7). In veterinary practice, the increased demand for products that might improve and accelerate the quality of the healing process is the topic of several medical and economic studies (2). Among these approaches, the application of platelet-rich plasma (PRP) or mesenchymal stem cells (MSCs) has been intensively investigated to promote the regeneration of a broad range of soft and hard tissues, including the skin (7). Autologous PRP is blood plasma that has been enriched with platelets through a specific centrifugation protocol. PRP is usually obtained from autologous blood and has been used to treat both acute and chronic skin wounds (4). Platelets are cytoplasmic fragments derived from bone marrow megakaryocytes and contain several types of growth factors and cytokines that are able to stimulate the wound healing process (1–3, 6–9). There are a number of *in vivo* studies in dogs and horses regarding the use of PRP for cutaneous wound therapy. There studies reported that during wound healing PRP promoted epithelial differentiation and skin regeneration (10–12). Mesenchymal stem cells (MSCs) are a population of adult stem cells particularly known for their paracrine activity, especially for their anti-inflammatory and pro-regenerative effects. Therefore, they might play a central role in wound healing, as they are able to induce cell proliferation, promote granulation tissue formation, and stimulate neovascularization (3, 6). Recent reports consider that both allogeneic and autologous MSCs may positively impact all phases of wound repair in humans as well as in animals; indeed, MSCs possess a low immunogenic profile allowing an allogeneic administration without risk of rejection (13, 14). There are few available studies using a combination of autologous MSC and PRP in animals (2–9). Regarding this, the use of PRP as clinical-grade adjuvant to enhance the therapeutic effectiveness of engrafted MSCs has been suggested by several studies, highlighting that PRP treatment improves the angiogenic potential of MSCs both *in vitro* and *in vivo*, while stimulating the proliferation of MSCs *in vitro* (7). However, systematic studies on whether PRP alters the repair properties of engrafted MSCs in skin wound healing are rarely reported (7, 15). The aim of this paper was to evaluate the quality of the healing process in experimentally induced full-thickness skin lesions treated with autologous PRP alone or in association with peripheral blood-derived mesenchymal stem cells (MSCs) in a large animal model.

## Materials and methods

### Animal model and ethical statement

Three female Bergamasca sheep (*Ovis aries*) homogeneous for size and age were included in this experimental study. Sheep were allocated in an experimental stable (MAPS Department, University of Padova, Legnaro, Italy) for 2 weeks before starting the experimentation to allow acclimation. Biochemistry and parasitological examinations were carried out to assess their health status prior to beginning the study. Ethical approval for the experiment was obtained by ministerial decree n° 51/2015-PR released by the Italian Ministry of Health (n°51/2015-PR), in accordance with the Body for the Protection of Animals (OPBA).

Sheep represent a good experimental model because they provide peculiar opportunities as a pre-clinical model for translational medicine (16). This is a small-scale study that should be considered as a preliminary investigation that aims to assess the effect of different innovative treatments on skin wound healing, which took into account the “3Rs” principle (replacement-reduction-refinement). The performed experiments complied with EU Directive 2010/63/EU for animal experimentation and at the end of the experimentation, sheep were relocated to a teaching farm instead of being sacrificed.

### Platelet-rich plasma preparation

Autologous PRP was obtained by using a double centrifugation tube method as described by Perazzi et al. (17) and described following published guidelines (18). In brief, a total volume of 25 mL blood (matched to lesion size and animal weight) was collected from the jugular vein using a commercially designed platelet sequestration sterile tubes containing sodium citrate (Vacutainer CPT; Becton, Dickinson and Company, Franklin Lakes, New Jersey, United States). After a first centrifugation (Labofuge 400, Heraeus Holding, Hanau, Germany) at 1300xg for 20 min without brakes at room temperature, the buffy coat containing mononuclear cells and platelets was collected and mixed in 0.75–1 mL of the remaining plasma. Then, different buffy coats from the same subject were pooled and centrifuged a second time at 300xg for 15 min without brakes at room temperature in order to obtain the platelet pellet. The pellet was collected and resuspended in the remaining volume of plasma. The whole procedure was carried out using sterile disposable tools. This procedure was performed on the day of surgery (day 0).

A complete hemogram of the PRP was carried out for each sheep using a flow cytometry hematology system (ADVIA 120 Analyzer, Bayer Lab, New York, United States). The final volume of PRP contained a mean value of 850 ± 185 ×10^3^ platlets/μL, a concentration four to five times greater than the basal level; it was used alone or as vehicle to administer the cell treatment.

In addition, the final product was investigated for the presence of three growth factors involved in different biological processes, including wound healing. The concentration was assessed by enzymatic-linked immunosorbent assay (ELISA) for insulin-like growth factor 1 (IGF-1), vascular endothelial growth factor A (VEGF-A), and transforming growth factor beta 1 (TGF-β1) following the manufacturer’s protocol (Supplementary Figure S1).

### Isolation of peripheral blood-derived mesenchymal stem cells

MSCs were isolated from three sheep that were not involved in the current experimentation. 100 mL of peripheral blood were collected from the jugular vein of each animal using a vacutainer coated with Li-heparin (Becton, Dickinson and Company, Franklin Lakes, New Jersey, United States), in order to avoid the formation of blood clots. MSCs were isolated following the protocol described by Martinello et al. (19). In brief, the blood was diluted 1:1 with phosphate buffered saline (PBS) solution and added onto a Ficoll-paque solution (Amersham Biosciences, Little Chalfont, United Kingdom) to obtain mononuclear cells (including MSCs) in the interphase after a density gradient centrifugation. Next, MSCs were cultured in a complete medium constituted of DMEM High Glucose (SigmaAldrich, St. Louis, MO, United States) supplemented with fetal bovine serum (FBS, 10%) (Euroclone, Milano, Italy) and antibiotics (1% penicillin–streptomycin; Euroclone, Milano, Italy). Cells were kept in an incubator at 37°C with 5% CO2. On the day of application, cells were detached from the tissue culture flasks using trypsin–EDTA 0.05% (Euroclone, Milano, Italy), counted, and diluted in PRP for the therapeutic application. Cell viability was checked along with counting using the trypan blue dye; all cells showed a viability ≥95%.

### Surgical procedure and experimental design

On the day of surgery, animals were treated with antibiotic (amoxicilline, 15 mg/Kg) and analgesic therapy (tramadol, 4 mg/Kg) via intramuscular injection. Afterwards, sheep were premedicated and sedated by administering medetomidine (0.01 mg/Kg) intravenously. After a proper level of sedation, the animal was placed in sternal decubitus position and the thoracolumbar area was shaved (trichotomy) for the preparation of the operative field. The induction of anesthesia was induced by administering propofol (4 mg/Kg). Afterwards to maintain it, the animal was orotracheally intubated and administered with isoflurane combined with a mixture of medical air and oxygen. Following this, the back of each animal was marked using a sterilized square model (a template), which was used as a guide to designate the wound areas to perform the creation of the lesion. Moreover, this step was also performed in order to take into account skin contraction during wound healing for a representative biopsy collection. After scrubbing the surgical field with iodine-povidone (10%), six full-thickness skin defects (16 cm^2^, 4 cm × 4 cm) were created on the back of each sheep using a scalpel and a sterilized square guide model. The wounds were equally distant from each other (6 cm ipsilateral and 5 cm contralateral) and the distance between them did not affect the healing process or the outcomes of the experiment (20, 21). Five lesions were used to assess the effects of five different treatments. The sixth lesion was used as control, in which only PBS was administered. The application of the five different therapies and the untreated (control) wound were randomized for each sheep. In this study, only the effects of PRP alone and the combination of PRP with MSCs were evaluated and compared to control wounds (PBS). Other treatments, which were not related to the application of PRP, are mentioned in other original research manuscripts (14, 22). The application of MSCs alone in the same animal model has been reported by Martinello and colleagues (23).

A total volume of 2 mL of PRP was applied to the PRP treatment wound: 1 mL was dropped on the wound surface including on the edge of the wound to fully cover the area of the lesion whereas the remnant 1 mL was subcutaneously injected in the wound margins homogeneously (i.e., 0.25 mL per margin). Wounds intended to be treated with PRP + MSC were managed in the same way. Briefly, a final concentration of 4×10^6^ MSCs diluted in in PRP was applied topically (1 mL) and subcutaneously injected in the wound borders (1 mL). The treatment was applied only once on the day of surgery. After the surgery, the lesions were bandaged using a sterile gauze. The sterile gauzes were further covered with a protective bandage and a tubular mesh gauze, the latter was fixed to the peripheral wool to secure the bandages to cover the wounds. Animals were all housed in an adequate barn to ensure their wellbeing as gregarious animals. Antibiotic therapy (amoxicilline, 150 mg/Kg; subcutaneous injection) was administered for 5 days and analgesic therapy (buprenorphine, 100 mcg/Kg; intramuscular injection) for 2 days. At 7, 14, 21, and 42 days after wounding, two skin samples for each lesion were collected using a 6-mm punch biopsy after proper sedation and analgesic therapy. Skin samples were collected at four different time points in order assess the wound healing process throughout all its stages. At each time point, samples were collected in two different precise points of the wounds that were opposite to each other and equidistantly located from the original wound margins (day 0) using the previously made marking points as a guide. For each time point, one biopsy was used for histopathological and immunohistochemistry analysis, and one for molecular analysis.

### Clinical follow-up

The wound dressings were changed every 3 days until the end of the study (day 42). In order to replace them, non-woven gauzes were wet with a sterile saline physiological solution to avoid damage to the healing wounds and impair the healing process. The macroscopic aspect of each lesion was documented with photographs taken at 7, 14, 21, and 42 days after surgery. This operation and the clinical evaluation were carried out by the same blinded operator at each time point. The percentages of wound re-epithelialization and contraction were quantified and calculated using an image processing program (ImageJ^®^).

### Histopathological analysis and immunohistochemistry

Nine biopsies per each time point (3 for each treatment and 3 for untreated wounds, for a total of 36) were processed for histopathological evaluation. Skin biopsies were fixed in neutral-buffered formalin (10%) for 24 h. Next, they were dehydrated with a gradual dilution scale of ethanol and embedded in paraffin following a standard protocol. After embedding, skin samples were cut using a microtome (Leica-RM2035, Leica Microsystems, Wetzlar, Germany) into 4-μm thick slices. For histopathological examination, skin sections were stained with hematoxylin and eosin (H&E) following the standard procedure. Then, slides were observed using a light microscope (Olympus Vanox AHBT3, Olympus, Tokyo, Japan). All sections were evaluated by two blinded independent operators for the following histopathological parameters: dermal and subcutaneous inflammation, immature granulation tissue (presence and development), and skin adnexa (presence and development). In order to evaluate these parameters, a semi-quantitative score from 0 to 3 (0 absence, 1 mild, 2 moderate, and 3 abundant) was used and assigned to each skin section for all parameters. All data were calculated for each subject and parameter, and presented as relative frequencies. Moreover, the epidermal thickness index (ETI) was calculated to determine the degree of epidermal hypertrophy after wounding and healing. In order to calculate the ETI, the average thickness of skin samples epidermis was calculated at 0 and 42 days after surgery. The epidermis thickness was measured in the 10 randomly selected fields for each sample (400x magnification). Then, the ETI was calculated based on an equation described by Rahmani-Neishaboor et al. (24): ETI = (epidermal thickness at day 42/epidermal thickness at day 0). Values equivalent to 1 represent a fully healed skin wound without scar formation, whereas values >1 indicate a newly formed hypertrophic epidermis. For immunohistochemistry, skin sections were immunostained with monoclonal antibodies for Ki67 (a nuclear marker for cell proliferation; 1:50; clone MIB-1, Dako, Santa Clara, CA, United States) and alpha smooth muscle actin (α-SMA, marker for the detection of myofibroblasts; 1:1000; clone 1A4, Dako, Santa Clara, CA, United States), following the manufacturer’s protocol. Briefly, after a proper deparaffinization and dehydration protocol, sections were treated by immersion in a 0.3% H_2_O_2_ solution (methanol) for 20 min to block endogenous peroxidase activity, thus preventing the development of aspecific signal (false positive staining). For the Ki67 immunostainings only, sections were exposed for antigen retrieval (heat induced epitope retrieval, HIER) by the use of 10 mM sodium citrate buffer (pH 6) at 95°C for 20 min. Afterwards, nonspecific binding sites in each section were blocked by the exposure to 2.5% normal goat serum for 1 h at room temperature. Then, sections were incubated with the primary antibody following a different step: for Ki67, sections were incubated overnight at +4°C, while α-SMA for 1 h at room temperature. After incubation, sections were washed three times with PBS and incubated with the secondary antibody (goat anti-mouse biotin conjugated IgG; 1:200; Dako, Santa Clara, CA, United States) for 30 min at room temperature. Following this, an avidin-biotin complex (ABC Reagent, VECTASTAIN^®^ ABC Kit, PK-4000, Vector Laboratories, Burlingame, CA, United States) and 3,3′-diamobenzidine (DAB) system (ImmPACT^®^ DAB Substrate, SK-4105, Vector Laboratories, Burlingame, CA, United States) were used to develop and obtain a proper immunolabeling. Counterstaining with Mayer’s hematoxylin was also performed. For assessing the specificity of the immunostaining reaction, positive and negative controls were always performed. Sections were observed using a light microscope (Olympus Vanox AHBT3, Olympus, Tokyo, Japan). The positive area of Ki67 was quantitatively analyzed: the percentage of the positive area was calculated in ten randomly selected fields of view (400x magnification) for each section. The positive signal was measured using an image processing program (ImageJ^®^), and the results are expressed as percentage of positive area. The assessment of the immunoreactivity of α-SMA was performed by using a semi-quantitative histological score (0–3) based on the abundancy and orientation of myofibroblasts as follows: 0 for absence of immunolabeling, 1 for a mild presence of immunoreaction and irregular orientation, 2 for moderate immunoreaction and well-oriented myofibroblasts, and 3 for abundant immunoreaction and compact parallel organized myofibroblasts.

### Gene expression analysis

Nine biopsies per time point (3 for treated and 3 for untreated wounds, for a total of 36) were processed for gene expression analysis by real time PCR (RT-PCR). After sampling, samples were snap-frozen in liquid nitrogen and stored at −80°C until use. RNA isolation was performed by using TRIzol reagent (Life Technologies, Carlsbad, CA, United States). Total RNA was quantified and assess for its quality (260/280 nm wavelengths ratio) using a NanoDrop™ spectrophotometer (Thermo Scientific, Waltham, MA, United States). Then, a total amount of 2 μg of RNA was retrotranscribed using the Superscript™ II Reverse Transcriptase (Invitrogen, Carlsbad, CA, United States) to obtain the complementary DNA (cDNA). The obtained cDNA was used as a template for RT-PCR analysis using the ABI 7500 Real Time PCR system (Applied Byosistems, Foster City, CA, United States). The relative expression of genes involved in wound healing were assessed: collagen type I (collagen 1α1, Col1α1), collagen type III (collagen 3α1, Col3α1), vascular endothelial growth factor A (VEGF-A), and hair-Keratin (hKER). The obtained data were normalized on the gene expression of the housekeeping gene 18S ribosomial RNA (18S) and Ribosomial Protein S24 (RPS24). The specific pair of primers for each gene were designed based on the sheep annotated genome sequence on the GenBank database (sheep genome assembly: Oar_v4.0, GCA_000298735.2) by using the Primer Express 3.0 software (Applied Byosystems, Foster City, CA, United States). The designed primers were validated by using the standard curve method, along with the calculation of their efficiency. Each pair of primers presented with an adequate slope (between −3.3 and −3.6) and with a respective efficiency of 95–100%. The efficiency was calculated using the ABI 7500 System SDS Software (Applied Biosystems, Foster City, CA, United States). For the relative quantification, all experiments were run in triplicate for each gene of interest. At the same time, the melting curve analysis (dissociation curve) was performed to detect non-specific products of amplification. The relative quantification was calculated with the 2^-ΔΔCt^ method. Uninjured skin of the animals involved in the current study was used as the calibrator sample.

### Statistical analysis

Data are expressed as mean ± standard error of the mean (SEM). Statistical differences were evaluated by using the two-way ANOVA test with statistical differences (*p* ≤ 0.05) detected by the Tukey’s “post-hoc” test. To evaluate the statistical differences regarding the ETI score, a Student’s t distribution was used and a *p* value less or equal to 0.05 was considered statistically significant. All statistical analysis were performed using GraphPad Prism 9.0 software (San Diego, CA, United States). Comparison among groups regarding the granulation tissue was performed using the Kruskal-Wallis non-parametric test by time using the XLSTAT software package (Data Analysis and Statistical Solution for Microsoft Excel, Addinsoft, Paris, France).

## Results

### Clinical follow-up

**Figure 1:**
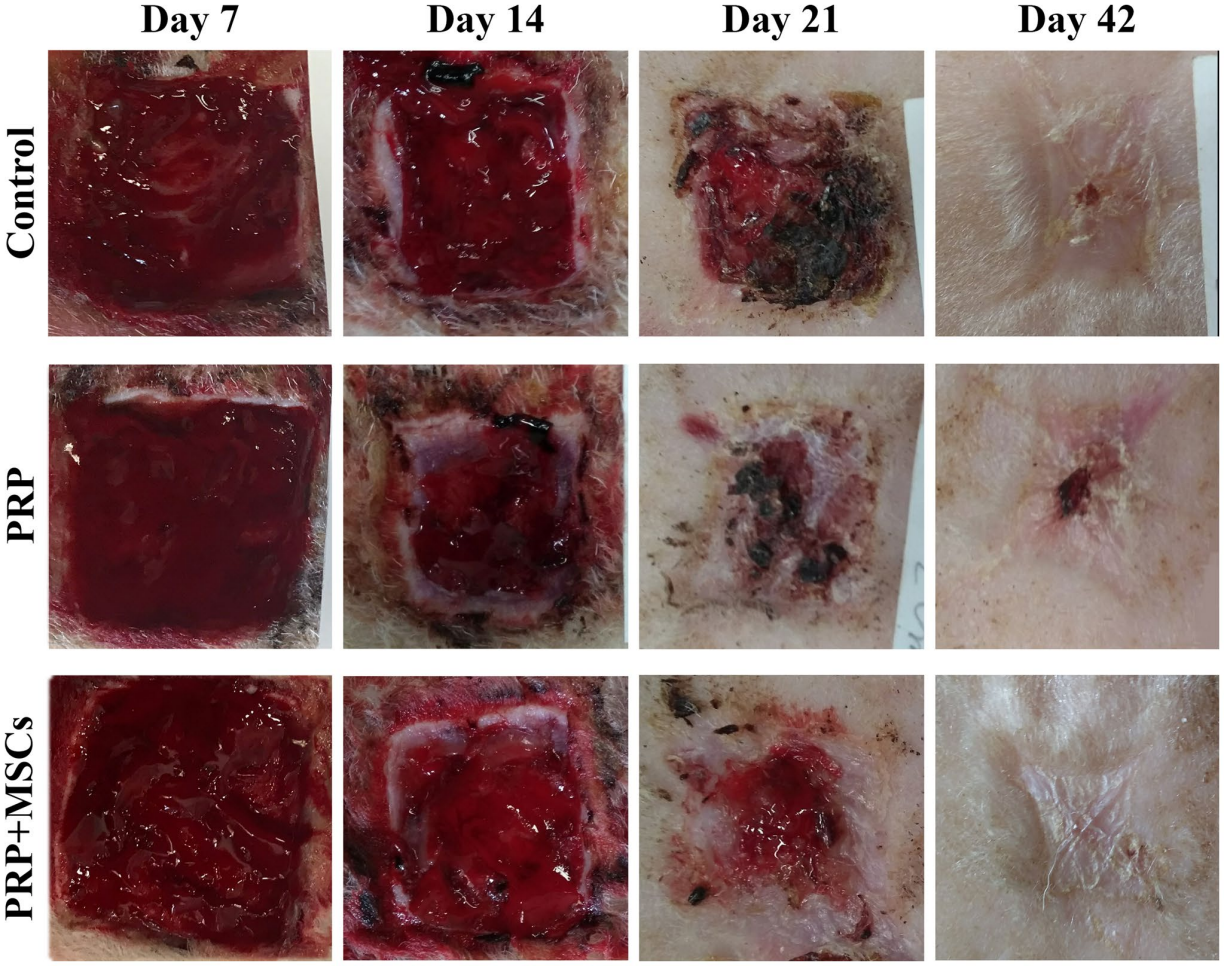
Representative macroscopic images of the skin wounds during the wound healing process.

The macroscopic appearance of the lesions was documented with photographs taken at 7, 14, 21, and 42 days after surgery using a ruler for measuring the evaluation of healing process (Figure 1). The presence of the granulation tissue, along with wound contraction and re-epithelialization rate were evaluated weekly until the complete closure of the wounds. The percentage of the wound contraction and re-epithelialization were calculated for each wound based on the formulas already reported in the literature (25, 26).

#### Granulation tissue

**Figure 2:**
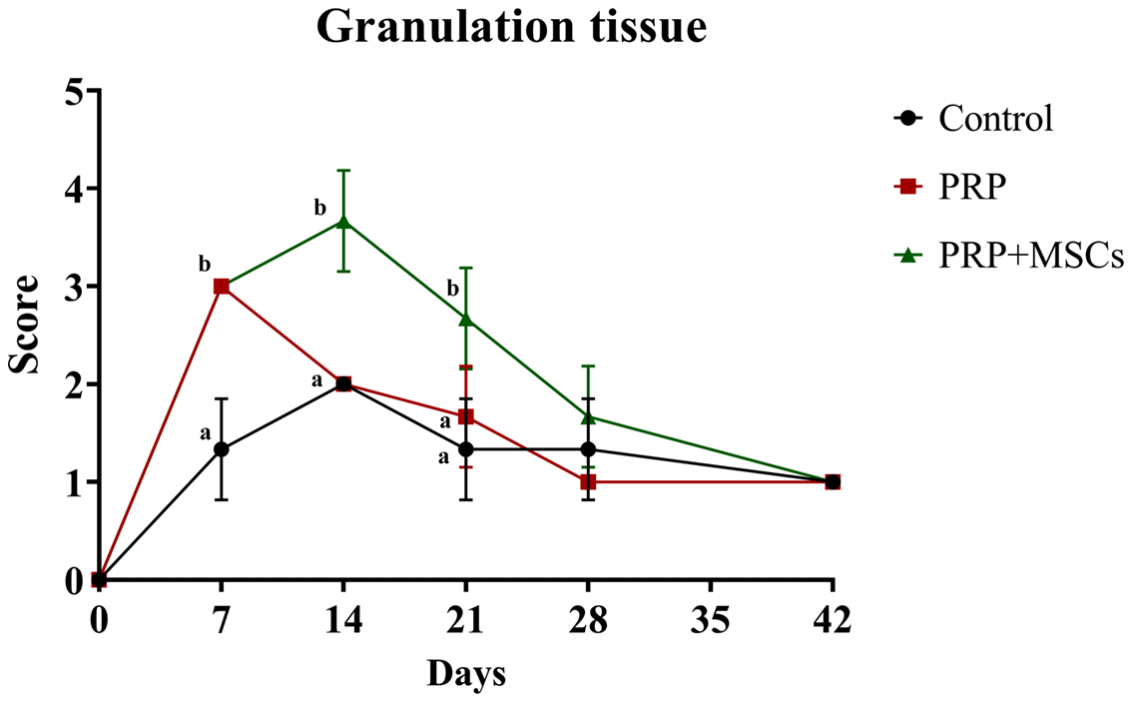
Clinical score for the presence of granulation tissue. Data are shown as mean ± SD. Different letters within time points means statistically significant different values for *p* < 0.05.

Overall, the greatest presence of granulation tissue was found around day 14 for all lesions, and then decreased until it disappeared in all lesions around day 42; notably, during the first 2 weeks in the lesions treated with PRP + MSC on days 7 and 14, there was a greater presence of granulation tissue (considered exuberant) than in the others, which is statistically significant compared to control wounds: day 7, 14, and 21 (*p* < 0.05) (Figure 2).

#### Wound contraction rate

**Figure 3:**
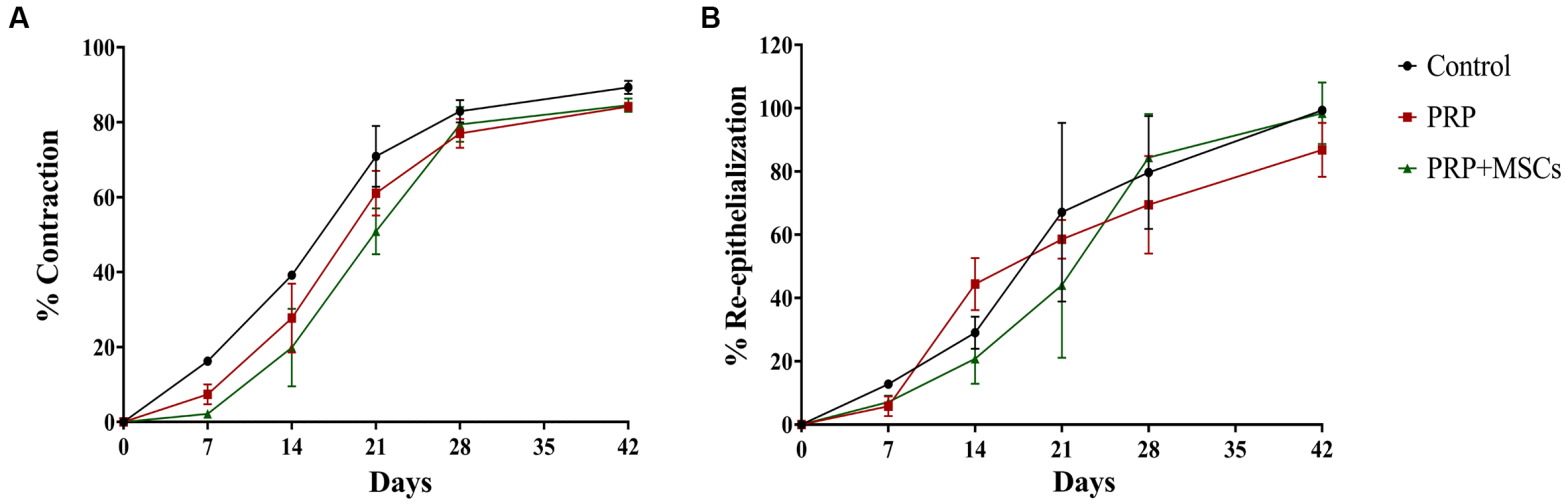
**(A)** Percentage of contraction and **(B)** re-epithelialization of skin wounds during the experimentation. Data are shown as mean ± SEM.

The biggest differences were between day 7 and day 28. In particular, on day 7 the control lesions already exceeded 16.25% of contraction while the PRP + MSCs was the least contracted at 2.18%. The PRP had a mean value of 7.37%. These differences are statistically significant (*p* < 0.001). On day 14 there was the greatest difference between the control values reaching about 39.18% and the PRP + MSCs reaching 19.87%. PRP reached values of 27.72%. All these differences are statistically significant (*p* = 0.024). On day 21 the control was the most contracted, reaching 70.91%, while the PRP + MSCs was less, reaching 50.87%. The PRP had a mean value reaching 61.06%. Between day 21 and 28 the rate of contraction of lesions treated with PRP + MSCs increased, reaching values similar to the others, and all lesions were between 77 and 79% (PRP and PRP + MSCs) and 82.95% (control) of contraction. At the end point (42 days) the percentage of contraction was between 84% (both in lesions treated with PRP and PRP + MSCs) and 89.33% in control lesions. Notably, throughout the healing period the contraction percentages were higher in the control lesions than in PRP and PRP + MSCs lesions. Moreover, the rate of contraction of lesions treated with PRP + MSCs was more constant than for lesions treated with PRP in which the contraction slowed down in the second part of the trial (Figure 3A).

#### Re-epithelialization rate

Contrary to the percentage of contraction, the average percentage values of re-epithelialization maintained different trends and values until day 42. Re-epithelialization appeared from day 7 with values between 5.77% (PRP) and 12.77% (control). By day 14, lesions treated with PRP reached 44.42%, control lesions 29.06% and finally PRP + MSCs 20.83%; these differences between PRP vs. PRP + MSCs are statistically significant (*p* = 0.019). At day 21 lesions treated with PRP + MSCs reached 44.06%, while those with PRP reached 58.53% and the control reached 67.10%. Between day 14 and day 21, there was the largest percentage increase for control lesions, while lesions treated with PRP underwent a slowdown. On day 28, PRP + MSC reached 84.42%, PRP 69.49% and control 79.67%. From day 21 to day 28 there was a significant increase in the percentage of epithelialization for PRP + MSC. At day 42 none of the treated lesions had healed completely: PRP + MSC reached a value of 94.40% and control 99.31% epithelialization. The lesions treated with PRP reached an average of 86.84% of epithelialization, showing a strong slowdown from day 14 (in which they were the most epithelialized) onwards. The data obtained indicate that PRP induced a high percentage of epithelialization between days 7 and 14 and then dropped drastically; meanwhile, PRP + MSCs induced a high rate of epithelialization between days 21 and 28. The control, on the other hand, had a more linear trend (Figure 3B).

### Histopathological observations

**Figure 4:**
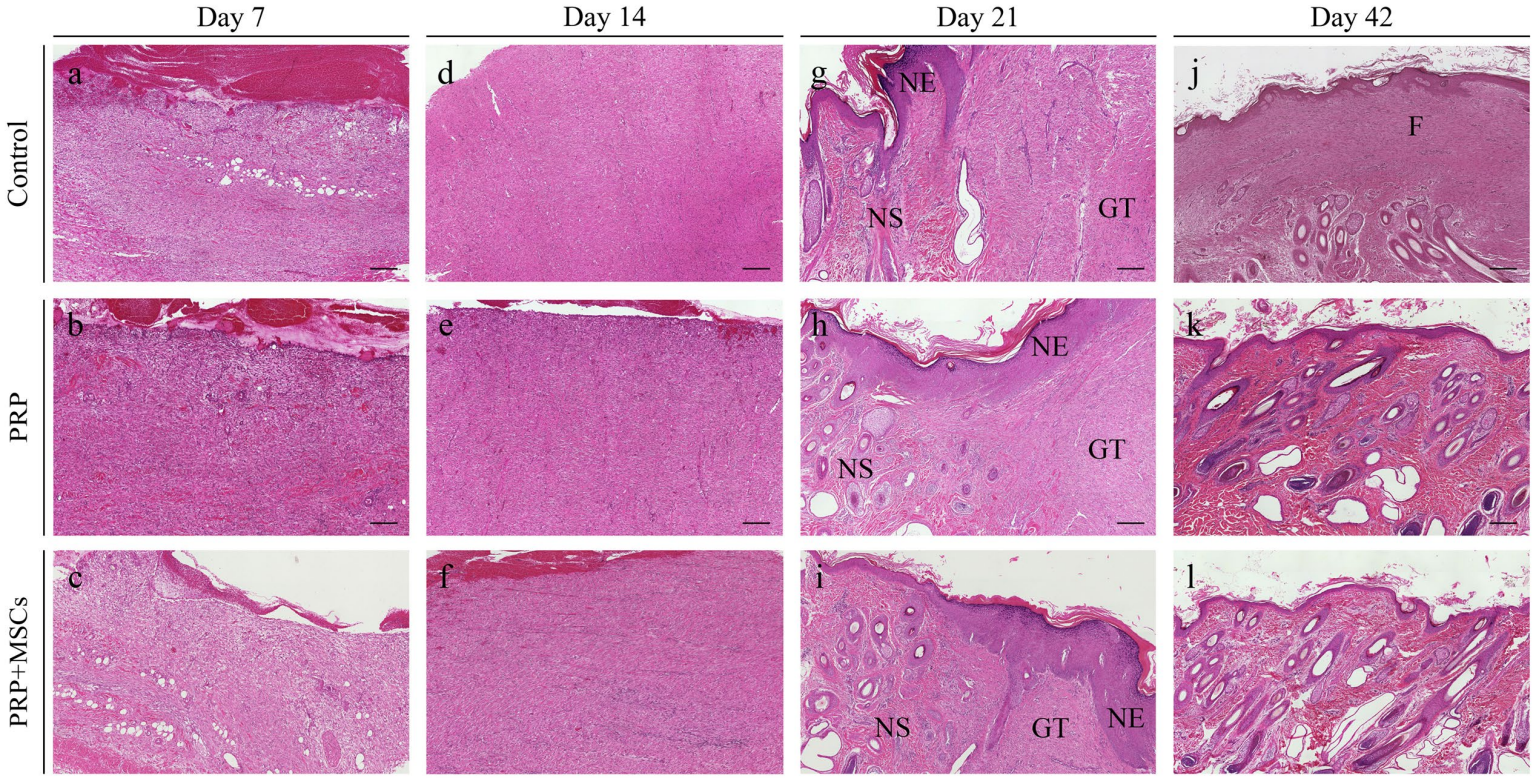
Histopathological microphotographs of the skin biopsies obtained at different time points during the wound healing process. **(A–C)** Skin wounds at 7 days; **(D–F)** wounds at 14 days; **(G–I)** wounds at 21 days; **(J–L)** wounds at 42 days after wounding. GT, granulation tissue; NE, neoepidermis; NS, neoskin; F, fibrosis. Scalebar, 200 μm.

**Figure 5:**
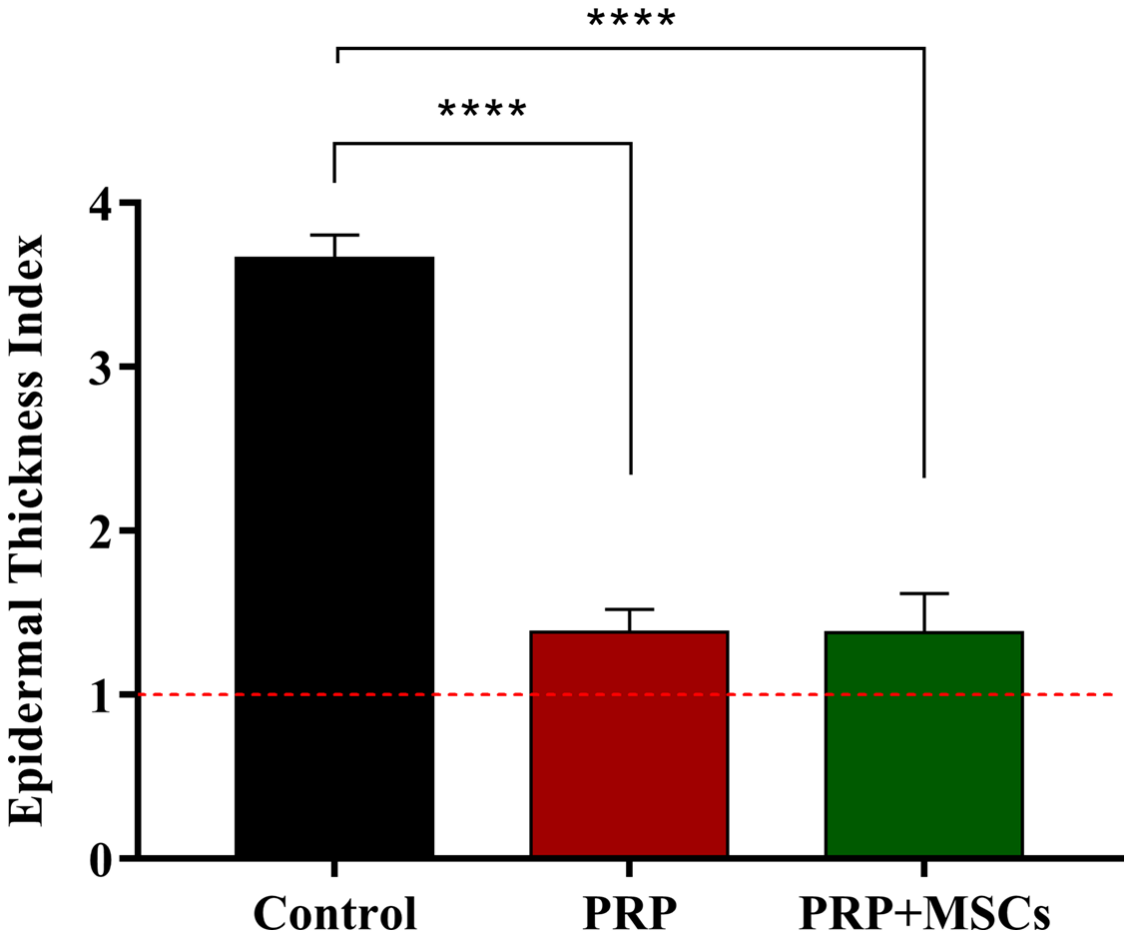
Epidermal thickness index (ETI) at 42 days of all wounds respect to unwounded skin. Data are shown as mean ± SEM; **** *p* < 0.0001.

*Superficial inflammation* – At day 7, all PRP-treated and untreated (control) wounds showed a mild superficial inflammation (100%) (Figures 4A,B) while PRP + MSCs wounds presented with a 33% with a moderate inflammation and the remnant wounds with a mild one (67%) (Figure 4C) as observed for other treatments. After two weeks, wounds treated with PRP showed the same frequency observed during the first week (100% mild superficial inflammation) (Figure 4E); on the other hand, all PRP + MSCs wounds showed a moderate inflammation (100%) (Figure 4F). At the same time point, untreated wounds presented with 37% of the wounds with a moderate inflammation and a 67% with a mild one (Figure 4D). At 21 days, all treated wounds (PRP and PRP + MSCs) showed no inflammation in the superficial layer of the dermis (100% absent) (Figures 4H,I), whereas a 33% of control wounds still presented with a mild inflammation and in 67% was absent (Figure 4G). At 42 days, all wounds showed no signs of superficial inflammation (100% absent) (Figures 4J–L). *Deep inflammation* – One week after wounding, all wounds showed a mild inflammation in the deeper layer of the dermis (100% mild) (Figures 4A–C). Between 14 and 21 days, the presence of deep inflammation was unaltered in PRP + MSCs-treated and control wounds (100% mild) (Figures 4D,F,G,I); on the contrary, at the same time points, PRP-treated wounds showed a reduction of inflammation in 33% of wounds (absent) while in 67% the inflammation was still considered mild (Figures 4E,H). At 42 days, all treated wounds did not show any element of inflammation (100% absent) (Figures 4K,L); regarding control wounds, the 100% presented with a mild inflammation was observed during previous time points (Figure 4J). *Immature granulation tissue* – Both treatments led to a more abundant deposition of granulation tissue (GT) at 7 days than control wounds (67% moderate and 33% mild in treated wounds vs. 67% mild and 33% absent in control wounds) (Figures 4A–C). At 2 weeks, PRP-treated wounds presented with a 100% abundant presence of granulation tissue (Figure 4E), PRP + MSCs wounds with a 100% moderate presence (Figure 4F), while control wounds showed a lower amount of GT (Figure 4D): 67% moderate and 33% mild. At 21 days, the GT started to reduce its amount and develop into a mature dermis as observed in treated wounds. 67% of the wounds treated with PRP showed a moderate presence of GT while in 33% it was absent (Figure 4H); on the other hand, wounds treated with PRP + MSCs showed a mild presence of GT in 67% while in 33% it was absent (Figure 4I). The presence of GT in control wounds was still considered moderate in 67% of the wounds and mild in the remaining 33% (Figure 4G). At 42 days, the GT was absent in all wounds (100%) (Figures 4J–L). *Skin adnexa* – The presence of skin adnexa was first observed at 21 days in all wounds, but at different frequencies (Figures 4A–F). The treatment with PRP led to a moderate presence of skin adnexa in all wounds (100%) (Figure 4H), while the application of PRP combined with MSCs led to 67% of wounds with an abundant presence and 33% a moderate amount of skin adnexa (Figure 4I). 100% of control wounds showed a mild presence of skin adnexa (Figure 4G). After 6 weeks, both treatments led to an abundant presence of skin adnexa in all wounds (100%) (Figures 4K,L). However, in wounds treated with PRP + MSCs, a better organization and development of hair follicles and apocrine glands was observed with respect to the treatment with PRP alone. On the contrary, in untreated wounds 67% presented with a moderate and 33% with an abundant amount of skin adnexa (Figure 4J). *Epidermal Thickness Index (ETI)* – Control wounds showed a higher ETI than both control groups (3,660 ± 0,145 control vs. 1,381 ± 0,142 PRP vs. 1,377 ± 0,241 PRP + MSCs); these differences were considered statistically significant (Figure 5). The application of PRP and PRP + MSCs led to a less thick epidermis compared to control wounds, resembling the one observed in unwounded skin (day 0).

### Immunohistochemical observations

#### Ki67

**Figure 6:**
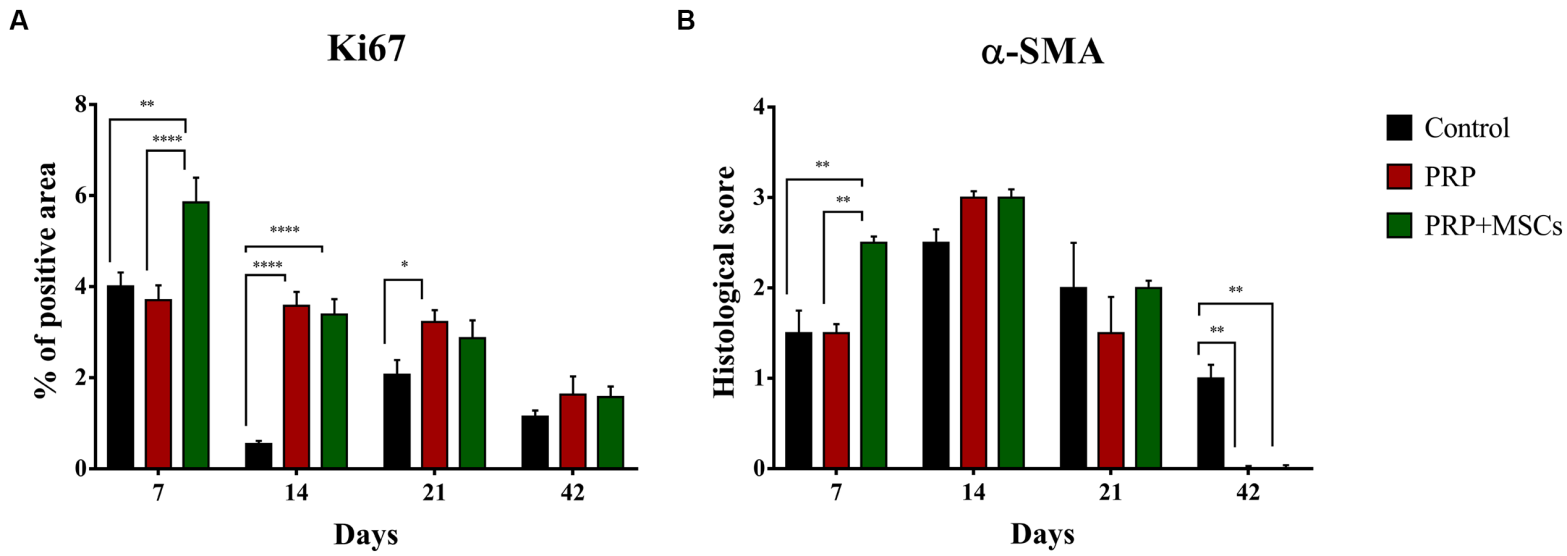
**(A)** Quantitative analysis of the percentage of positive area of each wound for the proliferation marker Ki67 and **(B)** histological score for the alpha smooth muscle actin (α-SMA). Data are shown as mean ± SEM; * *p* < 0.05; ** *p* < 0.01; *** *p* < 0,001; **** *p* < 0.0001.

Ki67 is considered a reliable marker to assess cell proliferation during the wound healing process (27, 28) (Figure 6A; Supplementary Figure S2). At 1 week, the combined treatment of PRP + MSCs induced cell proliferation compared to PRP alone and control wounds, which presented a similar percentage of positive area for Ki67 immunolabeling (5,849 ± 0,546 PRP + MSCs vs. 3,702 ± 0,327 PRP vs. 4,008 ± 0.304 control); these differences were statistically significant. At 14 days, both treatments presented with a similar amount of positivity, higher respect to control wounds (3,390 ± 0,336 PRP + MSCs vs. 3,579 ± 0,310 PRP vs. 0,546 ± 0,072 control). At this time point, both PRP alone and PRP + MSCs presented a significant statistically higher positive area than the control wounds. As observed after 2 weeks, on day 21 the application of PRP alone or in combination with MSCs stimulated cell proliferation, resulting in a higher positivity in treated wounds compared to control wounds (2,868 ± 0,394 PRP + MSCs vs. 3,225 ± 0,264 PRP vs. 2,068 ± 0,323 control). However, only the difference between PRP and control wounds was statistically significant. At day 42, all wounds presented with a similar percentage of positivity.

#### α-SMA

Myofibroblasts are a specialized cell population with a key role in wound contraction and deposition of components of the extracellular matrix during the wound healing process (29) (Figure 6B; Supplementary Figure S3). Immunopositivity for α-SMA was detected since day 7 in all wounds, especially in PRP + MSCs-treated wounds. Indeed, on this time point, these wounds presented a higher number of positive cells (i.e., myofibroblasts) along with a better organization (i.e., cells were parallel to the wound surface) compared to wounds treated with PRP alone or left untreated. These observed differences were statistically significant. On day 14, treated wounds (PRP and PRP + MSCs) presented with a slightly higher immunopositivity and better organization of cell when compared to control wounds. After 3 weeks, a reduced amount of α-SMA positive cells was observed in all wounds, especially in PRP-treated wounds. At 42 days, immunopositivity for α-SMA was observed in only untreated wounds in the superficial layer of the dermis. In treated wounds, no myofibroblasts were observed; the presence of immunolabeling in these samples at this time point has to be ascertained to arrector pili muscles and myoepithelial cells of apocrine glands.

### Gene expression analysis

**Figure 7:**
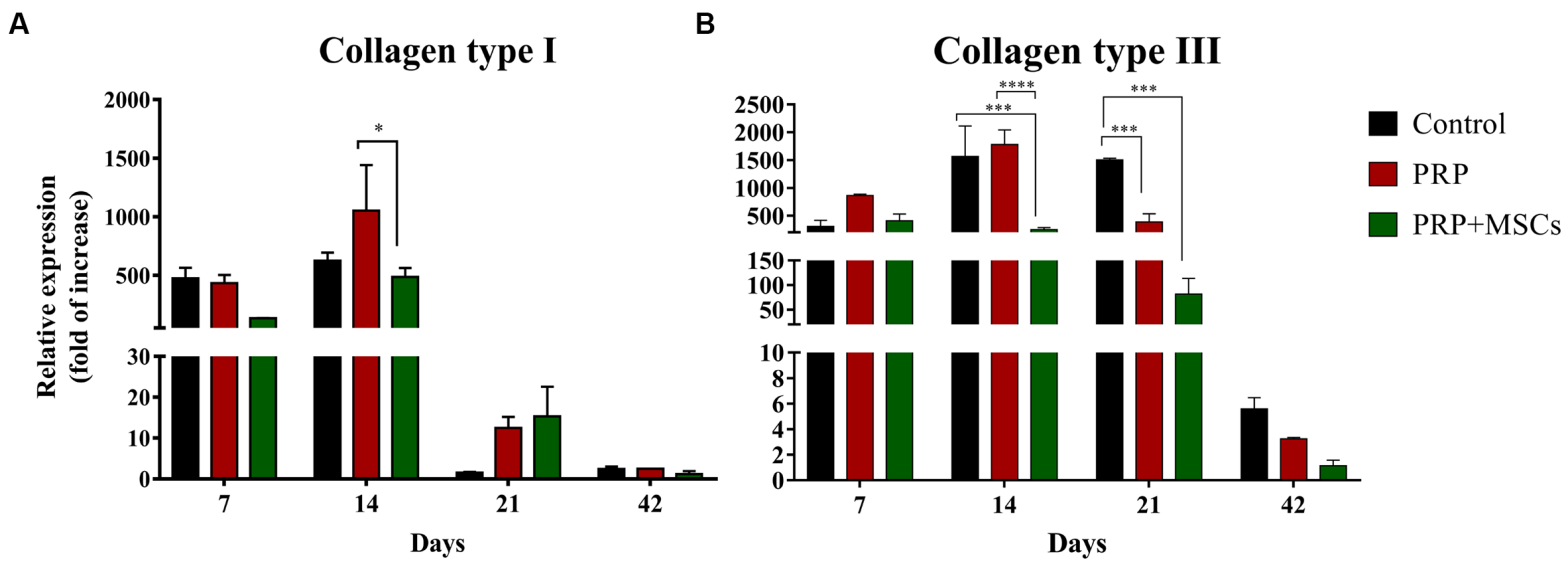
Real Time PCR for collagen type I and III. **(A)** Relative expression of collagen type I (Col1α1) gene and **(B)** collagen type III (Col3α1) gene at 7, 14, 21, and 42 days post-wounding. Data are shown as mean ± SEM. Unwounded skin was used as the calibrator sample. Statistical differences were measured between the three experimental groups at the same time point. * *p* < 0.05; *** *p* < 0.001; **** *p* < 0.0001.

**Figure 8:**
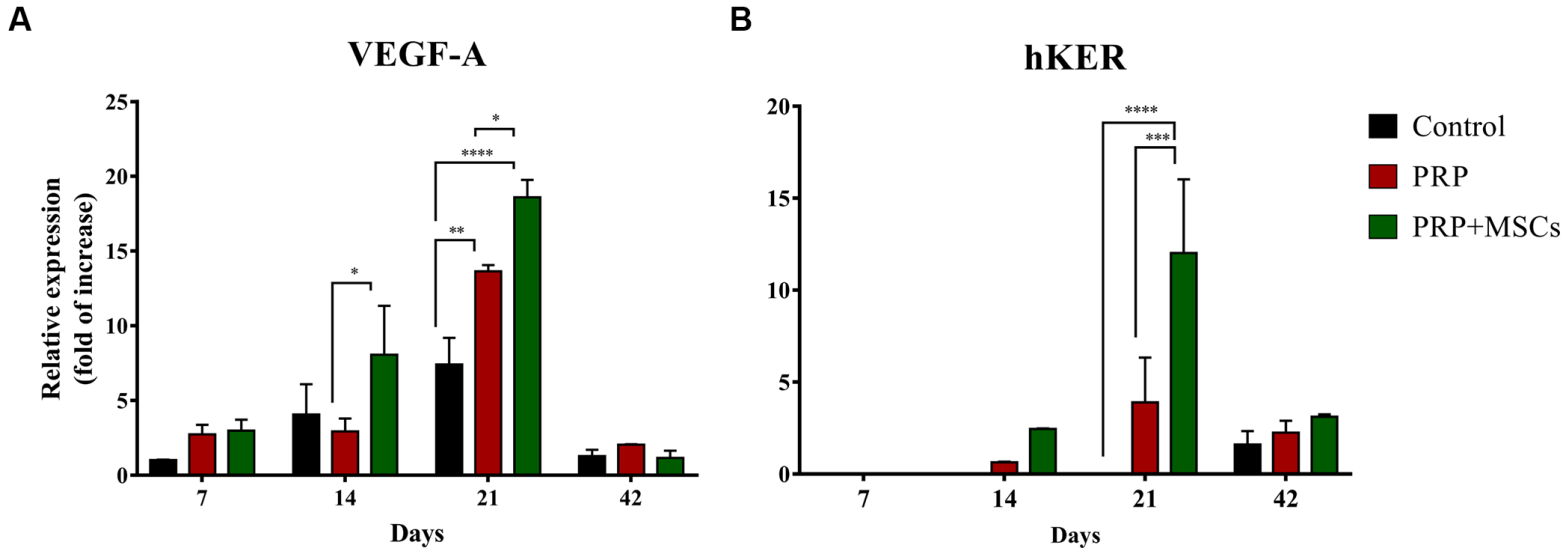
Real Time PCR for vascular endothelial growth factor A (VEGF) and hair-Keratin (hKER) genes. **(A)** Relative gene expression of the VEGF-A and **(B)** hKER at 7, 14, 21, and 42 days after wounding. Data are shown as mean ± SEM. Unwounded skin was used as the calibrator sample. Statistical differences were measured between the two experimental groups at the same time point; * *p* < 0.05; ** *p* < 0.01; *** *p* < 0.001; **** *p* < 0.0001.

RT-PCR analysis showed that the gene expression of both collagen types (I and III) was up-regulated in all wounds during the first 2 weeks (Figures 7A,B). At 7 days, the gene expression of collagen I (Col1α1) was slightly higher in control and PRP-treated wounds compared to wounds treated with PRP + MSCs. After 2 weeks, regarding the gene expression in untreated wounds and in those treated with PRP + MSCs, the observed mRNA level was similar while wounds treated with PRP presented with a higher gene expression compared to the other two groups; the difference between the two treatments was statistically significant. Regarding the gene expression analysis of collagen type III (Col3α1), at 1 week post-wounding all wounds presented a similar level of mRNA. At day 14, PRP-treated wounds along with control wounds presented a higher gene expression compared to wounds treated with the combined therapy, both in a statistically significant fashion. At 21 days, the gene expression of Collagen type I dropped in all wounds; this was seen in particular in control wounds where the observed mRNA level was almost absent. On the contrary, the relative expression of collagen type III was still high in control wounds. Treated wounds presented with a lower gene expression than the previous time points. After 6 weeks, the observed mRNA of both collagen type was lowered in all wounds. The mRNA level of VEGF-A, a fundamental growth factor involved in the neoangiogensis, was higher in wounds treated with the combined therapy at 7, 14, and 21 days (Figure 8A). In particular, at 14 days, the difference of relative expression observed between PRP + MSCs-treated wounds and PRP treatment alone was statistically significant. Meanwhile, at 3 weeks post-wounding, the difference was also statistically significant between PRP + MSCs-treated wounds and PRP alone, but also with control wounds; in addition, the difference between the PRP group and control wounds was significant. The gene expression of hKER, a keratin protein involved in the development of hair follicles, was first detected at day 14 in both treated groups, presenting a slightly higher mRNA level in wounds treated with the combined therapy (Figure 8B). At 21 days, both treated groups showed an upregulation of the relative expression of hKER. At the end of the experimentation (42 days), a low gene expression of hKER was also detected in control wounds for the first time in 6 weeks.

## Discussion

Skin wound healing is a complex and dynamic process consisting of overlapping phases, which has the final aim of restoring the physiological skin barrier with a newly formed tissue. However, in different clinical scenarios (e.g., diabetes, large extended wound, infection, etc.), an impairment may occur in any of its phases affecting the quality of tissue healing such as delaying wound closure or eventually leading to the formation of pathological scars. Furthermore, this affects the properties of the newly formed skin, which presents a lower quality in terms of biomechanical properties and appearance compared to the skin before wounding. Concomitantly, it could result in the progression of the wound into a chronic non-healing ulcer causing discomfort to the patient. Apart from concerning the welfare of the patient, wounds that inadequately heal also have consequences on wound care management with a subsequent impact in the economic sphere (30–33). For this reason, since conventional treatments are usually associated with a poor prognosis, it is necessary to investigate innovative therapies that might allow skin wounds to properly heal into a tissue characterized by similar pre-wound structural and functional properties.

In the current study, we describe the application of autologous platelet-rich plasma (PRP) in an experimental second intention wound healing large animal model and its combination with allogeneic mesenchymal stem cells (MSCs) to assess if these treatments are able to lead to a newly formed healed tissue that qualitatively is the result of tissue regeneration (*restitutio ad integrum*) rather than repair (scarring). The wound healing model was previously optimized (20, 23). Wounds were sufficiently distant between each other to prevent their interaction via the blood flow; hence, it was optimal to effectively distinguish between the effects of different treatments (21).

Inflammation is the first phase of wound healing, and it is a crucial step for the whole process. If on one hand, inflammatory cells allow the progression of healing by clearing the wounds from cellular debris and pathogens, on the other hand a sustained and persistent inflammation may slow down the whole healing consequently leading to wound chronicity (34, 35). In this study, inflammation and inflammatory infiltrate were evaluated at the histopathological level. During the first 2 weeks, a more intense inflammatory activity was observed in treated wounds compared to later stages. This event might be related to an earlier activation of the inflammation phase, and one reason might be the activation of platelets contained in the PRP after interaction with the collagen of the wound tissue (36), which might have resulted in an efficient release of growth factors from the granules contained in platelets. These different soluble mediators (e.g., chemokines, PDGF, etc.,) are able to attract inflammatory cells to the wound site, thus explaining the higher amount observed in treated wounds respect to control wounds during the first weeks of healing (37, 38). Indeed, the recruitment of inflammatory cells into the wound is essential for tissue debridement and inflammatory cells, such as macrophages, can release growth factors that may sustain the following phases of healing (39). However, in treated wounds the inflammatory process started to decrease around 21 days after wounding, and then disappearing completely after 6 weeks. Contrarily, untreated wounds showed a mild persistent inflammation at day 42, in particular in the deeper dermal layer of the skin. Although MSCs and PRP promoted a higher inflammatory response at the beginning, they later constrained it in the final stages of wound healing (6). Different studies have demonstrated the immunosuppressive properties of MSCs and their effect on local inflammation. MSCs secrete different molecules (PGE2, IL-10, etc.) that inhibit inflammatory cells proliferation and subsequently downregulate the production of pro-inflammatory cytokines as previously observed in studies regarding MSCs application for wound healing (5, 13, 40).

As the inflammations subsides, wounds that heal by second intention begin to cover the wound bed by the deposition of a provisional extracellular matrix known as granulation tissue. This immature extracellular matrix is characterized by a high cellularity and vascularization along with playing the important role of acting as a scaffold for migrating cells repopulating the wounded skin (41). Treated lesions, especially with the combined treatment PRP + MSCs, showed the greatest presence of granulation tissue. From day 7 to day 28 granulation tissue was more abundant than in PRP-treated lesions and, especially, compared to control; moreover, it regressed later than in other lesions. Along with the rapid onset, there was also a greater persistence of the granulation tissue compared to PRP and control group during the first weeks of healing. As a matter of fact, a higher histological presence of granulation tissue was also observed in treated wounds compared to untreated ones during the first 2 weeks. Even if granulation tissue deposition was faster and moderate in treated wounds, it never led to an exuberant presence in any lesion, whereas in control wounds it showed a slower deposition and development, with an abundant presence during the first 2 weeks. The higher deposition in treated wounds could be attributed to the presence of PRP and MSCs. These two products might have promoted cell proliferation and the deposition of extracellular matrix in the wounds as previously observed (6, 23, 42). These observations are also supported by the higher immunopositivity of Ki67 in treated wounds, a well-known nuclear marker expressed by proliferating cells (27, 28). Indeed, PRP + MSCs-treated wounds presented with a higher presence of proliferating cells at day 7 respect to PRP alone. On the other hand, untreated lesions always showed a lower level of cells in active proliferation, and this might be reflected in the slower deposition and maturation of the granulation tissue. The positive and rapid effect of platelets, observed in PRP-treated and PRP + MSCs-treated wounds, on the healing mechanisms is linked to the release of growth factors and cytokines that have a role in fibroblasts proliferation and migration along with stimulating production of extracellular matrix (43–45). However, the duration of their time-limited action could be due to the short half-life of platelets and might require repeated administration of exogenous growth factor (1), which was not performed in the current study. Similarly, MSCs are a well-known stem cell population for their broad release of growth factors (TGF-β1, bFGF, IGF-1, PDFG, etc.) that might have supported, and enhanced, the PRP effect (as observed during the first week): namely, cell proliferation and deposition of proteins involved in the formation of granulation tissue extracellular matrix. These clinical results suggest the use of PRP + MSC in subacute or chronic wounds in humans and animals that are characterized by scarcity of granulation tissue, and therefore with poor healing tendency as already well recognized for PRP (12, 43, 45–48) and only rarely reported in the literature for PRP + MSC (49).

Another important factor contributing to the development and maturation of granulation tissue is angiogenesis (50, 51). In fact, the presence of vessels in the wound is fundamental to allow the distribution of nutrients and oxygen to the healing skin, hence sustaining the healing process. During the first 2 weeks of this study, an increase of the mRNA levels of VEGF-A was observed in lesions treated with the combined treatment (PRP + MSCs). Moreover, at day 21 both treated groups showed a higher gene expression of VEGF-A mRNA. The greater presence of this growth factor in treated wounds than in control wounds might have sustained an increased presence of neoangiogenesis and tissue reperfusion, thereby supporting a faster maturation and resolution of granulation tissue as observed histologically in the treated wounds. In accordance with this result, both PRP and MSCs release several growth factors that can support and boost the formation of new vessels such as bFGF and PDGF (52–54), and the addition of MSCs to the PRP might have enhanced its effect.

Furthermore, a key role in the development of the granulation tissue is played by myofibroblasts. This specialized cell population, differentiated from local fibroblasts, is characterized by the expression of a-SMA and are the main effectors of wound contraction (29). This process is fundamental for healing as it diminishes the wound area by pulling its edges to the center of the lesion (55). At the same time, myofibroblasts can also secrete components of the ECM, which are fundamental for the development of granulation tissue. Regarding the contraction rate, it should be noted that throughout healing (in particular between day 7 and day 28) in both lesions treated with PRP, but especially those treated with both PRP and MSCs, a lower percentage of contraction than in untreated wounds was observed. These results affirm what is reported in the literature (56). This data could indicate that healing occurs qualitatively in a manner more similar to tissue regeneration than the reparative mechanism of lesions control with higher rates of contractions (57). Furthermore, the rate of contraction of lesions treated with PRP + MSCs is more constant than in lesions treated with PRP, in which the contraction slowed down in the second part of healing. Histologically at day 7, lesions treated with PRP + MSCs presented a higher presence of myofibroblasts (α-SMA immunopositivity) with respect to only PRP-treated wounds and untreated ones. After 2 weeks, the levels of α-SMA positive cells increased in all wounds but were higher in both treated wounds compared to untreated wounds. This result might be ascribed to the presence of TGF-β1 in the PRP, which is known to induce the differentiation of fibroblasts into myofibroblasts (5). MSCs as well can release TGF-β1, and this might explain the higher presence of α-SMA-positive cells at day 7 in combined treated wounds as the higher presence of these factor, from both PRP and MSCs, could have induced an early differentiation of fibroblasts, hence the more abundant presence of myofibroblasts at that time point in PRP + MSCs-treated wounds (1, 56).

Nevertheless, the higher histological score for α-SMA in treated wounds at 14 days was mainly related to the better organization of myofibroblasts, and not to the cell number, as they were aligned in parallel with respect to the wound surface; on the contrary, myofibroblasts in control wounds appeared randomly organized. A proper organization of myofibroblasts should result in a more efficient, but not faster, wound contraction (58–60), compared to unaligned myofibroblasts as in untreated wounds. Indeed, the contraction rate was higher in the latter during the first weeks after wounding. In these terms, treatment did not influence the contraction rate but rather provided proper biomechanical properties to the healing tissue at the expense of the wound contraction rate. At the same time points (day 7 and 14), all wounds presented with a high gene expression of collagen type I and III, with the latter being fundamental for the development of the granulation tissue (41). In particular, at day 14 PRP-treated wounds showed the highest expression of collagen type III while lesions treated with the combined treatment had the lowest mRNA level among wounds. This result is in accordance with literature as PRP is known to stimulate the production of collagen in skin wound healing (5, 40, 61) and MSCs can secrete bioactive factors with anti-fibrotic properties thus controlling the expression of collagen genes (53, 62). Despite this difference in collagen type III gene expression between PRP alone and in combination with MSCs, both wounds showed an orderly arrangement of collagen also dictated by myofibroblasts orientation as they are usually co-aligned with collagen fibrils (63); this characteristic was maintained throughout the healing process. This is fundamental as collagen fibers’ arrangement determines the quality of the last phase of healing: tissue remodeling (58, 64).

As fibroblasts start to migrate to the wound site and generate the granulation tissue, keratinocytes from the wound border start to migrate as well to cover the wound (65). In particular, lesions treated with PRP showed a strong increase in re-epithelialization between days 7 and 14 (in which exceeded 44%) and in the second half of healing they decreased drastically compared to other treatments and control lesions. This result could be related to the half-life of platelets which is about 10 days. It may suggest the need to perform a second treatment of PRP in the lesion to allow a lengthening of the effect of platelets after their physiological platelet apoptosis (66, 67), as already demonstrated in dogs (12). In contrast, in lesions treated with PRP + MSCs the re-epithelialization rate increased greatly between day 21 and 28. These results are in agreement with what is reported in the literature and may be due to the fact that PRP, in addition to exerting an anti-fibrotic, pro-angiogenic and favoring proliferation and re-epithelialization action (68–70), is able to increase the migration, proliferation and survival of MSCs due to the action of TGF-β1 and the inhibitory effect on caspase-3 (56, 71). MSCs are able to anticipate the onset of the proliferative phase through upregulation of growth factors such as EGF, TGF-β1 and stromal-derived growth factor-1α, and to shorten the inflammatory phase thanks to the downregulation of TNF-α (23). Therefore, the association of MSCs and PRP might have a potent synergistic effect by significantly increasing TGF-β1 concentration and angiogenesis compared to individual treatments (56); this growth factor is also involved in inducing the differentiation of suprabasal cells of the epidermal layer, hence stimulating re-epithelialization and regeneration (5). Simultaneously, an increase in the duration of the proliferative phase and re-epithelialization and a decrease in contraction and processes of fibrosis and scarring have also been observed (56, 72–74). In skin lesions of mice and dogs treated with the association of PRP and MSCs, a lower deposition of fibroblasts has been reported thanks to the action of MSCs and a significant increase in the presence of skin adnexa at the site of lesion compared to lesions subjected to individual treatments (74, 75). This synergy between treatments is also reported in other studies carried out on bone, tendon and cartilage tissue lesions in dog and humans, which describe a substantial improvement in vascularization and orientation of fiber arrangement compared to lesions treated only with PRP or MSC (73, 76–78). Our clinical results showing a reduction in fibrosis and contraction, and an increase in re-epithelialization and cell proliferation are in agreement with what is reported in the literature and lead us to believe that the combined treatment with PRP + MSC has a positive effect on the quality of the healing process and is able to achieve healing qualitatively in a manner more similar to tissue regeneration.

In the later stages of healing, known as the remodeling or maturation phase, the granulation tissue begins to reorganize itself into a mature tissue (i.e., the dermis) by replacing its provisional matrix with structural ECM components such as collagen type I while reducing cellularity (41, 79). Starting from day 21, histologically all wounds presented different grades of maturation of the granulation tissue with the combined treatment showing a higher amount of loose ECM. This result was reflected on the collagen type I and III gene expression. The treated wounds, especially the PRP + MSCs-treated wounds, started to show a reduction of both collagens’ mRNA levels on day 21; untreated wounds showed a higher expression of collagen type III than the rest, whereas collagen type I gene expression was almost undetected in these lesions. This correlation between a higher presence of mature collagen fibers and PRP + MSCs treatment has been previously observed in animal study (80). The granulation tissue was resolved in all wounds at day 42.

Along with the maturation of the granulation tissue, skin adnexa started to appear, and their presence was particularly observed in wounds treated with PRP + MSCs and in a lesser amount in lesions PRP-treated alone. The presence of hair follicles might have been supported by the higher expression of hair keratin (hKER) observed in these wounds since day 14, and absent in untreated ones until day 42. At day 42, in PRP + MSCs-treated wounds a better organization and maturation of skin appendages was observed with respect to PRP-treated wound, in which hair follicles and skin glands were randomly organized. MSCs are known to possess positive effects on hair growth (81, 82) and might have stimulated the activation of stem cells in hair follicle bulge from the wound margins (83) and hair follicle development. Untreated lesions presented a lower number of skin appendages and in addition they still presented an area of the dermis immunopositive for α-SMA. This area also presented highly compact collagen fibrils organized in parallel to the skin surface. This observation, along with the exuberant gene expression of collagen type III at day 21 and absent expression of collagen type I, might have led to an excessive deposition of immature ECM and indicate that it might correspond to dermal fibrosis (84, 85), resembling the appearance of hypertrophic scars (86–88). This might also be the direct consequence of the observed lack of organization of collagen fibrils during the healing process; an orderly disposition of collagen fibers lowers the occurrence of healing by excessive scarring (64, 89). Moreover, the same wounds showed a thicker epidermis than treated wounds as observed by the ETI index; increased epidermal thickness and keratosis are among the other signs of pathological scarring (85). Thus, preventing the formation of excessive deposition of fibrotic tissue is crucial.

The current study presents two major limitations: the reduced number of animals and a weak data robustness due to the low number of subjects included in the study. For this reason, the study has to be considered as a preliminary study. However, the comforting exploratory results obtained push to progress in the studies and implement the number of subjects treated with the investigated innovative therapies.

## Conclusion

In this study, the application of PRP and its combination with allogeneic MSCs was investigated in a large animal wound healing model. The application of PRP accelerated the wound healing process, especially during the proliferative phase, by stimulating the deposition and maturation of the granulation tissue. PRP along with MSCs showed similar results but with a constant progression of the healing process. Even if less rapid during the first half of healing, the application of PRP + MSCs allowed a proper advancement of the healing process throughout its phases. Eventually, wounds treated with PRP and MSCs showed well-developed and organized collagen fibers and skin adnexa along with no dermal fibrosis. Overall, both treatments promoted and supported skin wound healing with the combined treatment of PRP and MSCs showing a qualitatively better structural organization of the repaired skin with features resembling a mature healthy skin.

## Data availability statement

The datasets presented in this study can be found in online repositories. The names of the repository/repositories and accession number(s) can be found in the article/Supplementary material.

## Ethics statement

The animal study was reviewed and approved by Body for the Protection of Animals (OPBA) with the executive order 116/92 and the ministerial decree n.51/2015-PR released by the Ministry of Italian Health.

## Author contributions

II, AP, MP, TM, and LM contributed to study design and supervised the study. II and AP performed surgery and collected clinical data. AC, TM, and LM performed the histopathological and gene expression analysis. BC and LM performed the statistical analysis. II, AP, and LM wrote the original draft of the manuscript. II, AP, MP, BC, TM, and LM revised and edited the manuscript. All authors contributed to the article and approved the submitted version.

## Funding

This work was supported by a grant from the University of Padova, Italy (BIRD:183588/18).

## Conflict of interest

The authors declare that the research was conducted in the absence of any commercial or financial relationships that could be construed as a potential conflict of interest.

## Publisher’s note

All claims expressed in this article are solely those of the authors and do not necessarily represent those of their affiliated organizations, or those of the publisher, the editors and the reviewers. Any product that may be evaluated in this article, or claim that may be made by its manufacturer, is not guaranteed or endorsed by the publisher.

## Supplementary material

Supplementary Figure 1 Growth factors quantification in Platelet-rich plasma (PRP): insulin-like growth factor 1 (IGF-1; CSB-E13753Sh, Cusabio, Aurogene Srl, Rome, Italy), vascular endothelial growth factor A (VEGF-A; Quantikine ELISA kit, DVE00, R&D Systems, Bio-Techne SRL, Milan, Italy), and transforming growth factor beta 1 (TGF-β1; TGF-beta 1 DuoSet ELISA, DY240, R&D Systems, Bio-Techne SRL, Milan, Italy). All experiments were run following the protocol provided by the manufacturer. Data are expressed as mean ± SEM.

Supplementary Figure 2 Microphotographs for Ki67 immunolabeling. **(A–C)** Skin wounds at 7 days; **(D–F)** wounds at 14 days; **(G–I)** wounds at 21 days; **(J–L)** wounds at 42 days after wounding. Scalebar = 200 μm.

Supplementary Figure 3 Microphotographs for alpha smooth muscle actin (α-SMA) immunostaining. **(A–C)** Skin wounds at 7 days; **(D–F)** wounds at 14 days; **(G–I)** wounds at 21 days; **(J–L)** wounds at 42 days after wounding. Scalebar = 200 μm.

## References

[1] Borena BM Martens A Broeckx SY Meyer E Chiers K Duchateau L. Regenerative skin wound healing in mammals: state-of-the-art on growth factor and stem cell based treatments. Cell Physiol Biochem. (2015) 36:1–23. doi: 10.1159/000374049 25924569

[2] Badis D Omar B. The effectiveness of platelet-rich plasma on the skin wound healing process: a comparative experimental study in sheep. Vet World. (2018) 11:800–8. doi: 10.14202/vetworld.2018.800-808, PMID: 30034173 PMC6048094

[3] Aydin O Karaca G Pehlivanli F Altunkaya C Uzun H Özden H. Platelet-rich plasma may offer a new Hope in suppressed wound healing when compared to mesenchymal stem cells. J Clin Med. (2018) 7:143. doi: 10.3390/jcm7060143, PMID: 29890683 PMC6025374

[4] Abdullaha BK Atasoyb N Omer AK. Evaluate the effects of platelet rich plasma (PRP) and zinc oxide ointment on skin wound healing. Ann Med Surg (Lond). (2019) 37:30–7. doi: 10.1016/j.amsu.2018.11.009, PMID: 30581567 PMC6297907

[5] Farghali HA AbdElKader NA Khattab MS AbuBakr HO. Evaluation of subcutaneous infiltration of autologous platelet-rich plasma on skin-wound healing in dogs. Biosci Rep. (2017) 37:BSR20160503. doi: 10.1042/BSR20160503, PMID: 28246352 PMC5469334

[6] Chicharro D Carrillo JM Rubio M Cugat R Cuervo B Guil S. Combined plasma rich in growth factors and adipose-derived mesenchymal stem cells promotes the cutaneous wound healing in rabbits. BMC Vet Res. (2018) 14:288. doi: 10.1186/S12917-018-1577-Y, PMID: 30241533 PMC6151009

[7] Hersant B Sid-Ahmed M Braud L Jourdan M Baba-Amer Y Paul Meningaud JP. Platelet-rich plasma improves the wound healing potential of mesenchymal stem cells through paracrine and metabolism alterations. Stem Cells Int. (2019) 2019:1234263. doi: 10.1155/2019/1234263 31781232 PMC6875194

[8] Blanton MW Hadad I Johnstone BH Mund JA Rogers PI Eppley BL. Adipose stromal cells and platelet-rich plasma therapies synergistically increase revascularization during wound healing. Plast Reconstr Surg. (2009) 123:56S–64S. doi: 10.1097/PRS.0b013e318191be2d, PMID: 19182664

[9] Argôlo Neto NM Del Carlo RJ Monteiro BS Nardi NB Chagastelles PC De Brito AFS. Role of autologous mesenchymal stem cells associated with platelet-rich plasma on healing of cutaneous wounds in diabetic mice. Clin Exp Dermatol. (2012) 37:544–53. doi: 10.1111/J.1365-2230.2011.04304.X 22712860

[10] Carter CA Jolly DG Worden CE Hendren DG Kane CJM. Platelet-rich plasma gel promotes differentiation and regeneration during equine wound healing. Exp Mol Pathol. (2003) 74:244–55. doi: 10.1016/S0014-4800(03)00017-0, PMID: 12782011

[11] DeRossi R Coelho ACAO De Mello GS Frazílio FO Leal CRB Facco GG. Effects of platelet-rich plasma gel on skin healing in surgical wound in horses. Acta Cir Bras. (2009) 24:276–81. doi: 10.1590/S0102-86502009000400006, PMID: 19705027

[12] Iacopetti I Patruno M Melotti L Martinello T Bedin S Badon T. Autologous platelet-rich plasma enhances the healing of large cutaneous wounds in dogs. Front Vet Sci. (2020) 7:575449. doi: 10.3389/FVETS.2020.575449, PMID: 33195571 PMC7649378

[13] Kim JW Lee JH Lyoo YS Jung DI Park HM. The effects of topical mesenchymal stem cell transplantation in canine experimental cutaneous wounds. Vet Dermatol. (2013) 24:242–e53. doi: 10.1111/VDE.12011, PMID: 23432413 PMC3618380

[14] Melotti L Martinello T Perazzi A Martines E Zuin M Modenese D. Could cold plasma act synergistically with allogeneic mesenchymal stem cells to improve wound skin regeneration in a large size animal model? Res Vet Sci. (2021) 136:97–110. doi: 10.1016/j.rvsc.2021.01.019 33596495

[15] Iacono E Merlo B Pirrone A Antonelli C Brunori L Romagnoli N. Effects of mesenchymal stem cells isolated from amniotic fluid and platelet-rich plasma gel on severe decubitus ulcers in a septic neonatal foal. Res Vet Sci. (2012) 93:1439–40. doi: 10.1016/j.rvsc.2012.04.008, PMID: 22579411

[16] Ribitsch I Baptista PM Lange-Consiglio A Melotti L Patruno M Jenner F. Large animal models in regenerative medicine and tissue engineering: to do or not to do. Front Bioeng Biotechnol. (2020) 8:2020. doi: 10.3389/fbioe.2020.00972 PMC7438731 32903631

[17] Perazzi A Busetto R Martinello T Drigo M Pasotto D Cian F. Description of a double centrifugation tube method for concentrating canine platelets. BMC Vet Res. (2013) 9:146. doi: 10.1186/1746-6148-9-146, PMID: 23876182 PMC3723642

[18] Harrison P Alsousou J. Studies on platelet rich plasma - new editorial policy for “platelets”. Platelets. (2020) 31:281–2. doi: 10.1080/09537104.2020.1729013 32124684

[19] Martinello T Bronzini I Perazzi A Testoni S De Benedictis GM Negro A. Effects of in vivo applications of peripheral blood-derived mesenchymal stromal cells (PB- MSCs) and platlet-rich plasma (PRP) on experimentally injured deep digital flexor tendons of sheep. J Orthop Res. (2013) 31:306–14. doi: 10.1002/jor.22205 22893604

[20] Broeckx SY Borena BM Van Hecke L Chiers K Maes S Guest DJ. Comparison of autologous versus allogeneic epithelial-like stem cell treatment in an in vivo equine skin wound model. Cytotherapy. (2015) 17:1434–46. doi: 10.1016/J.JCYT.2015.06.004, PMID: 26212608

[21] Liu X Barresi R Kaminer M Qian K Thillou F Bataillon M. Utilization of ex vivo tissue model to study skin regeneration following microneedle stimuli. Sci Rep. (2022) 12:18115–8. doi: 10.1038/s41598-022-22481-w, PMID: 36302808 PMC9613915

[22] Melotti L Martinello T Perazzi A Iacopetti I Ferrario C Sugni M. A prototype skin substitute, made of recycled marine collagen, improves the skin regeneration of sheep. Animals. (2021) 11:1–20. doi: 10.3390/ani11051219, PMID: 33922557 PMC8145883

[23] Martinello T Gomiero C Perazzi A Iacopetti I Gemignani F DeBenedictis GM. Allogeneic mesenchymal stem cells improve the wound healing process of sheep skin. BMC Vet Res. (2018) 14:202. doi: 10.1186/S12917-018-1527-8, PMID: 29940954 PMC6019727

[24] Rahmani-Neishaboor E Yau FM Jalili R Kilani RT Ghahary A. Improvement of hypertrophic scarring by using topical anti-fibrogenic/anti-inflammatory factors in a rabbit ear model. Wound Repair Regen. (2010) 18:401–8. doi: 10.1111/j.1524-475X.2010.00598.x, PMID: 20546553

[25] Kaufman T Levin M Hurwitz DJ. The effect of topical hyperalimentation on wound healing rate and granulation tissue formation of experimental deep second degree burns in guinea-pigs. Burns Incl Therm Inj. (1984) 10:252–6. doi: 10.1016/0305-4179(84)90003-2, PMID: 6713239

[26] Sardari K Kakhki EG Mohri M. Evaluation of wound contraction and epithelialization after subcutaneous administration of Theranekron in cows. Comp Clin Path. (2007) 16:197–200. doi: 10.1007/s00580-006-0657-8

[27] Lokmic Z Darby IA Thompson EW Mitchell GM. Time course analysis of hypoxia, granulation tissue and blood vessel growth, and remodeling in healing rat cutaneous incisional primary intention wounds. Wound Repair Regen. (2006) 14:277–88. doi: 10.1111/J.1743-6109.2006.00122.X, PMID: 16808806

[28] Sato T Abe T Ichioka S. Factors impairing cell proliferation in the granulation tissue of pressure ulcers: impact of bacterial burden. Wound Repair Regen. (2018) 26:284–92. doi: 10.1111/WRR.12675, PMID: 30265416

[29] Hinz B Gabbiani G. Fibrosis: recent advances in myofibroblast biology and new therapeutic perspectives. F1000 Biol Rep. (2010) 2:78. doi: 10.3410/B2-78, PMID: 21170369 PMC2998803

[30] Theoret CL. The pathophysiology of wound repair. Vet Clin North Am - Equine Pract. (2005) 21:1–13. doi: 10.1016/j.cveq.2004.11.001 15691596

[31] Riggs J Frazer Jennings JL Friend EJ Halfacree Z Nelissen P Holmes MA. Outcome of full-thickness skin grafts used to close skin defects involving the distal aspects of the limbs in cats and dogs: 52 cases (2005-2012). J Am Vet Med Assoc. (2015) 247:1042–7. doi: 10.2460/JAVMA.247.9.1042, PMID: 26480014

[32] Balsa IM Culp WTN. Wound care. Vet Clin North Am Small Anim Pract. (2015) 45:1049–65. doi: 10.1016/J.CVSM.2015.04.009 26022525

[33] Eggleston RB. Wound management: wounds with special challenges. Vet Clin North Am Equine Pract. (2018) 34:511–38. doi: 10.1016/J.CVEQ.2018.07.003 30447768

[34] Rosique RG Rosique MJ Farina Junior JA. Curbing inflammation in skin wound healing: a review. Int J Inflam. (2015) 2015:1–9. doi: 10.1155/2015/316235, PMID: 26356299 PMC4556061

[35] Menke NB Ward KR Witten TM Bonchev DG Diegelmann RF. Impaired wound healing. Clin Dermatol. (2007) 25:19–25. doi: 10.1016/J.CLINDERMATOL.2006.12.005 17276197

[36] Farndale RW. Collagen-induced platelet activation. Blood Cells Mol Dis. (2006) 36:162–5. doi: 10.1016/J.BCMD.2005.12.016 16464621

[37] Harrison S Vavken P Kevy S Jacobson M Zurakowski D Murray MM. Latelet activation by collagen provides sustained release of anabolic cytokines (2011) 39:729–34. doi: 10.1177/0363546511401576, PMC3176726 21398575

[38] Sonmez O Sonmez M. Role of platelets in immune system and inflammation. Porto Biomed J. (2017) 2:311–4. doi: 10.1016/J.PBJ.2017.05.005, PMID: 32258788 PMC6806752

[39] Nakai K. Multiple roles of macrophage in skin. J Dermatol Sci. (2021) 104:2–10. doi: 10.1016/J.JDERMSCI.2021.08.008 34493430

[40] Karayannopoulou M Papazoglou LG Loukopoulos P Kazakos G Chantes A Giannakas K. Locally injected autologous platelet-rich plasma enhanced tissue perfusion and improved survival of long subdermal plexus skin flaps in dogs. Vet Comp Orthop Traumatol. (2014) 27:379–86. doi: 10.3415/VCOT-14-02-0030, PMID: 25088504

[41] Häkkinen L Larjava H Koivisto L. Granulation tissue formation and remodeling. Endod Top. (2011) 24:94–129. doi: 10.1111/ETP.12008

[42] Stoff A Rivera AA Sanjib NB Moore ST Michael NT Espinosa-de-Los-Monteros A. Promotion of incisional wound repair by human mesenchymal stem cell transplantation. Exp Dermatol. (2009) 18:362–9. doi: 10.1111/j.1600-0625.2008.00792.x, PMID: 18803656 PMC2664391

[43] Crovetti G Martinelli G Issi M Barone M Guizzardi M Campanati B. Platelet gel for healing cutaneous chronic wounds. Transfus Apher Sci. (2004) 30:145–51. doi: 10.1016/J.TRANSCI.2004.01.004, PMID: 15062754

[44] Bennett NT Schultz GS. Growth factors and wound healing: biochemical properties of growth factors and their receptors. Am J Surg. (1993) 165:728–37. doi: 10.1016/S0002-9610(05)80797-4, PMID: 8506974

[45] Knighton DR Ciresi KF Fiegel VD Austin LL Butler EL. Classification and treatment of chronic nonhealing wounds. Successful treatment with autologous platelet-derived wound healing factors (PDWHF). Ann Surg. (1986) 204:322–30. doi: 10.1097/00000658-198609000-00011, PMID: 3753059 PMC1251286

[46] Ahmed M Reffat SA Hassan A Eskander F. Platelet-rich plasma for the treatment of clean diabetic foot ulcers. Ann Vasc Surg. (2017) 38:206–11. doi: 10.1016/j.avsg.2016.04.023, PMID: 27522981

[47] Suthar M Gupta S Bukhari S Ponemone V. Treatment of chronic non- healing ulcers using autologous platelet rich plasma: a case series. J Biomed Sci. (2017) 24:16–14. doi: 10.1186/s12929-017-0324-1, PMID: 28241824 PMC5327512

[48] Tambella AM Attili AR Dini F Palumbo Piccionello A Vullo C Serri E. Autologous platelet gel to treat chronic decubital ulcers: a randomized, blind controlled clinical trial in dogs. Vet Surg. (2014) 43:726–33. doi: 10.1111/J.1532-950X.2014.12148.X, PMID: 24484268

[49] Lian Z Yin X Li H Jia L He X Yan Y. Synergistic effect of bone marrow-derived mesenchymal stem cells and platelet-rich plasma in streptozotocin-induced diabetic rats. Ann Dermatol. (2014) 26:1–10. doi: 10.5021/AD.2014.26.1.1, PMID: 24648680 PMC3956772

[50] Carmeliet P Jain RK. Molecular mechanisms and clinical applications of angiogenesis. Nature. (2011) 473:298–307. doi: 10.1038/NATURE10144, PMID: 21593862 PMC4049445

[51] Landén NX Li D Ståhle M. Transition from inflammation to proliferation: a critical step during wound healing. Cell Mol Life Sci. (2016) 73:3861–85. doi: 10.1007/S00018-016-2268-0, PMID: 27180275 PMC5021733

[52] Lubkowska A Dolegowska B Banfi G. Growth factor content in PRP and their applicability in medicine. J Biol Regul Homeost Agents (2012) 26:3S–22S. Available at: https://europepmc.org/article/MED/23648195 (accessed April 7, 2023). 23648195

[53] Wu Y Chen L Scott PG Tredget EE. Mesenchymal stem cells enhance wound healing through differentiation and angiogenesis. Stem Cells. (2007) 25:2648–59. doi: 10.1634/STEMCELLS.2007-0226 17615264

[54] An T Chen Y Tu Y Lin P. Mesenchymal stromal cell-derived extracellular vesicles in the treatment of diabetic foot ulcers: application and challenges. Stem cell Rev reports. (2021) 17:369–78. doi: 10.1007/S12015-020-10014-9, PMID: 32772239

[55] Lesperance MM Francis TL Norton B. Postsurgical soft tissue healing. Postsurgical Orthop Sport Rehabil Knee Shoulder. (2006):3–18. doi: 10.1016/B978-032302702-1.50004-1

[56] Mahmoudian-Sani MR Rafeei F Amini R Saidijam M. The effect of mesenchymal stem cells combined with platelet-rich plasma on skin wound healing. J Cosmet Dermatol. (2018) 17:650–9. doi: 10.1111/JOCD.12512, PMID: 29504236

[57] Yannas IV Tzeranis DS So PTC. Regeneration of injured skin and peripheral nerves requires control of wound contraction, not scar formation. Wound Repair Regen. (2017) 25:177–91. doi: 10.1111/WRR.12516, PMID: 28370669 PMC5520812

[58] Gurtner GC Werner S Barrandon Y Longaker MT. Wound repair and regeneration. Nature. (2008) 453:314–21. doi: 10.1038/NATURE07039 18480812

[59] Schultz GS Davidson JM Kirsner RS Bornstein P Herman IM. Dynamic reciprocity in the wound microenvironment. Wound Repair Regen. (2011) 19:134–48. doi: 10.1111/J.1524-475X.2011.00673.X 21362080 PMC3051353

[60] Toriseva M Laato M Carpén O Ruohonen ST Savontaus E Inada M. MMP-13 regulates growth of wound granulation tissue and modulates gene expression signatures involved in inflammation, proteolysis, and cell viability. PLoS One. (2012) 7:e42596. doi: 10.1371/JOURNAL.PONE.0042596, PMID: 22880047 PMC3413640

[61] Xu P Wu Y Zhou L Yang Z Zhang X Hu X. Platelet-rich plasma accelerates skin wound healing by promoting re-epithelialization. Burn Trauma. (2020) 8:tkaa028. doi: 10.1093/BURNST/TKAA028, PMID: 32821743 PMC7427034

[62] Guillamat-Prats R. The role of MSC in wound healing, scarring and regeneration. Cells. (2021) 10:1729. doi: 10.3390/CELLS10071729, PMID: 34359898 PMC8305394

[63] Li S Van Den Diepstraten C D’Souza SJ Chan BMC Pickering JG. Vascular smooth muscle cells orchestrate the assembly of type I collagen via α2β1 integrin, RhoA, and fibronectin polymerization. Am J Pathol. (2003) 163:1045–56. doi: 10.1016/S0002-9440(10)63464-5, PMID: 12937145 PMC1868248

[64] Yang L Witten TM Pidaparti RM. A biomechanical model of wound contraction and scar formation. J Theor Biol. (2013) 332:228–48. doi: 10.1016/J.JTBI.2013.03.013, PMID: 23563057

[65] Ben Amar M Wu M. Re-epithelialization: advancing epithelium frontier during wound healing. J R Soc Interface. (2014) 11:20131038. doi: 10.1098/RSIF.2013.1038, PMID: 24451391 PMC3928935

[66] Mason KD Carpinelli MR Fletcher JI Collinge JE Hilton AA Ellis S. Programmed anuclear cell death delimits platelet life span. Cells. (2007) 128:1173–86. doi: 10.1016/J.CELL.2007.01.037, PMID: 17382885

[67] Sjaastad V Sand O Hove K. Physiology of Domestic Animals. 3nd ed. Oslo: Scandinavian Veterinary Press (2016).

[68] Jee CH Eom NY Jang HM Jung HW Choi ES Won JH. Effect of autologous platelet-rich plasma application on cutaneous wound healing in dogs. J Vet Sci. (2016) 17:79–87. doi: 10.4142/JVS.2016.17.1.79, PMID: 27051343 PMC4808647

[69] Lee H-W Reddy MS Geurs N Palcanis KG Lemons JE Rahemtulla FG. Efficacy of platelet- rich plasma on wound healing in rabbits. J Periodontol. (2008) 79:691–6. doi: 10.1902/jop.2008.070449 18380563

[70] Pereira RC Da F De La Côrte FD Brass KE da Silva Azevedo M Gallio M Cantarelli C. Evaluation of three methods of platelet-rich plasma for treatment of equine distal limb skin wounds. J Equine Vet Sci. (2019) 72:1–7. doi: 10.1016/J.JEVS.2017.10.009, PMID: 30929771

[71] Drengk A Zapf A Stürmer EK Stürmer KM Frosch KH. Influence of platelet-rich plasma on chondrogenic differentiation and proliferation of chondrocytes and mesenchymal stem cells. Cells Tissues Organs. (2009) 189:317–26. doi: 10.1159/000151290, PMID: 18689989

[72] Xie X Wang Y Zhao C Guo S Liu S Jia W. Comparative evaluation of MSCs from bone marrow and adipose tissue seeded in PRP-derived scaffold for cartilage regeneration. Biomaterials. (2012) 33:7008–18. doi: 10.1016/J.BIOMATERIALS.2012.06.058, PMID: 22818985

[73] Yoon JP Chung SW Kim JY Lee BJ Kim HS Kim JE. Outcomes of combined bone marrow stimulation and patch augmentation for massive rotator cuff tears. Am J Sports Med. (2015) 44:963–71. doi: 10.1177/0363546515625044, PMID: 26851271

[74] Zubin E Conti V Leonardi F Zanichelli S Ramoni R Grolli S. Regenerative therapy for the management of a large skin wound in a dog. Clin Case Reports. (2015) 3:598–603. doi: 10.1002/CCR3.253, PMID: 26273450 PMC4527804

[75] Formigli L Paternostro F Tani A Mirabella C Quattrini Li A Nosi D. MSCs seeded on bioengineered scaffolds improve skin wound healing in rats. Wound Repair Regen. (2015) 23:115–23. doi: 10.1111/WRR.12251, PMID: 25571903

[76] Abdel AL Aly A Menoufy HE Hassan A Ragae A Atta HM. Influence of Autologus adipose derived stem cells and PRP on regeneration of dehiscence-type defects in alveolar bone: A comparative histochemical and Histomorphometric study in dogs. Int J Stem Cells. (2011) 4:61–9. doi: 10.15283/ijsc.2011.4.1.61 24298335 PMC3840972

[77] Yamada Y Ueda M Naiki T Nagasaka T. Tissue- engineered injectable bone regeneration for osseointegrated dental implants. Clin Oral Implants Res. (2004) 15:589–97. doi: 10.1111/j.1600-0501.2004.01038.x, PMID: 15355402

[78] Yun S Ku SK Kwon YS. Adipose-derived mesenchymal stem cells and platelet-rich plasma synergistically ameliorate the surgical-induced osteoarthritis in beagle dogs. J Orthop Surg Res. (2016) 11:9. doi: 10.1186/S13018-016-0342-9, PMID: 26768536 PMC4714505

[79] Aravinthan A Park JK Hossain MA Sharmila J Kim HJ Kang CW. Collagen-based sponge hastens wound healing via decrease of inflammatory cytokines. 3. Biotech. (2018) 8:1–9. doi: 10.1007/S13205-018-1497-3/METRICS PMC6240018 30467532

[80] Pelizzo G Avanzini MA Icaro Cornaglia A Osti M Romano P Avolio L. Mesenchymal stromal cells for cutaneous wound healing in a rabbit model: pre-clinical study applicable in the pediatric surgical setting. J Transl Med. (2015) 13:219. doi: 10.1186/S12967-015-0580-3, PMID: 26152232 PMC4495634

[81] Bak DH Choi MJ Kim SR Lee BC Kim JM Jeon ES. Human umbilical cord blood mesenchymal stem cells engineered to overexpress growth factors accelerate outcomes in hair growth. Korean J Physiol Pharmacol. (2018) 22:555–66. doi: 10.4196/KJPP.2018.22.5.555, PMID: 30181702 PMC6115345

[82] Ou KL Kuo YW Wu CY Huang BH Pai FT Chou HH. The potential of a hair follicle mesenchymal stem cell-conditioned medium for wound healing and hair follicle regeneration. Appl Sci. (2020) 10:2646. doi: 10.3390/APP10082646

[83] Levy V Lindon C Zheng Y Harfe BD Morgan BA. Epidermal stem cells arise from the hair follicle after wounding. FASEB J. (2007) 21:1358–66. doi: 10.1096/FJ.06-6926COM, PMID: 17255473

[84] Xue M Jackson CJ. Extracellular matrix reorganization during wound healing and its impact on abnormal scarring. Adv Wound Care. (2015) 4:119–36. doi: 10.1089/WOUND.2013.0485/ASSET/IMAGES/LARGE/FIGURE4.JPEG, PMID: 25785236 PMC4352699

[85] Limandjaja GC Belien JM Scheper RJ Niessen FB Gibbs S. Hypertrophic and keloid scars fail to progress from the CD34−/α-smooth muscle actin (α-SMA)+ immature scar phenotype and show gradient differences in α-SMA and p16 expression. Br J Dermatol. (2020) 182:974–86. doi: 10.1111/BJD.18219, PMID: 31206605

[86] Slemp AE Kirschner RE. Keloids and scars: a review of keloids and scars, their pathogenesis, risk factors, and management. Curr Opin Pediatr. (2006) 18:396–402. doi: 10.1097/01.MOP.0000236389.41462.EF, PMID: 16914994

[87] Gauglitz GG Korting HC Pavicic T Ruzicka T Jeschke MG. Hypertrophic scarring and keloids: Pathomechanisms and current and emerging treatment strategies. Mol Med. (2011) 17:113–25. doi: 10.2119/MOLMED.2009.00153, PMID: 20927486 PMC3022978

[88] Wells A Nuschke A Yates CC. Skin tissue repair: matrix microenvironmental influences. Matrix Biol. (2016) 49:25–36. doi: 10.1016/J.MATBIO.2015.08.001, PMID: 26278492 PMC4753148

[89] Zeng XL Sun L Zheng HQ Wang GL Du YH Lv XF. Smooth muscle-specific TMEM16A expression protects against angiotensin II-induced cerebrovascular remodeling via suppressing extracellular matrix deposition. J Mol Cell Cardiol. (2019) 134:131–43. doi: 10.1016/J.YJMCC.2019.07.002, PMID: 31301303

