# Supplementary figures and images for: Assessment of the quality of the healing process in experimentally induced skin lesions treated with autologous platelet concentrate associated or unassociated with allogeneic mesenchymal stem cells: preliminary results in a large animal model
Source: Front Vet Sci. 2023 Jul 25;10:1219833. doi: 10.3389/fvets.2023.1219833 (PMC10407250; doi:10.3389/fvets.2023.1219833)

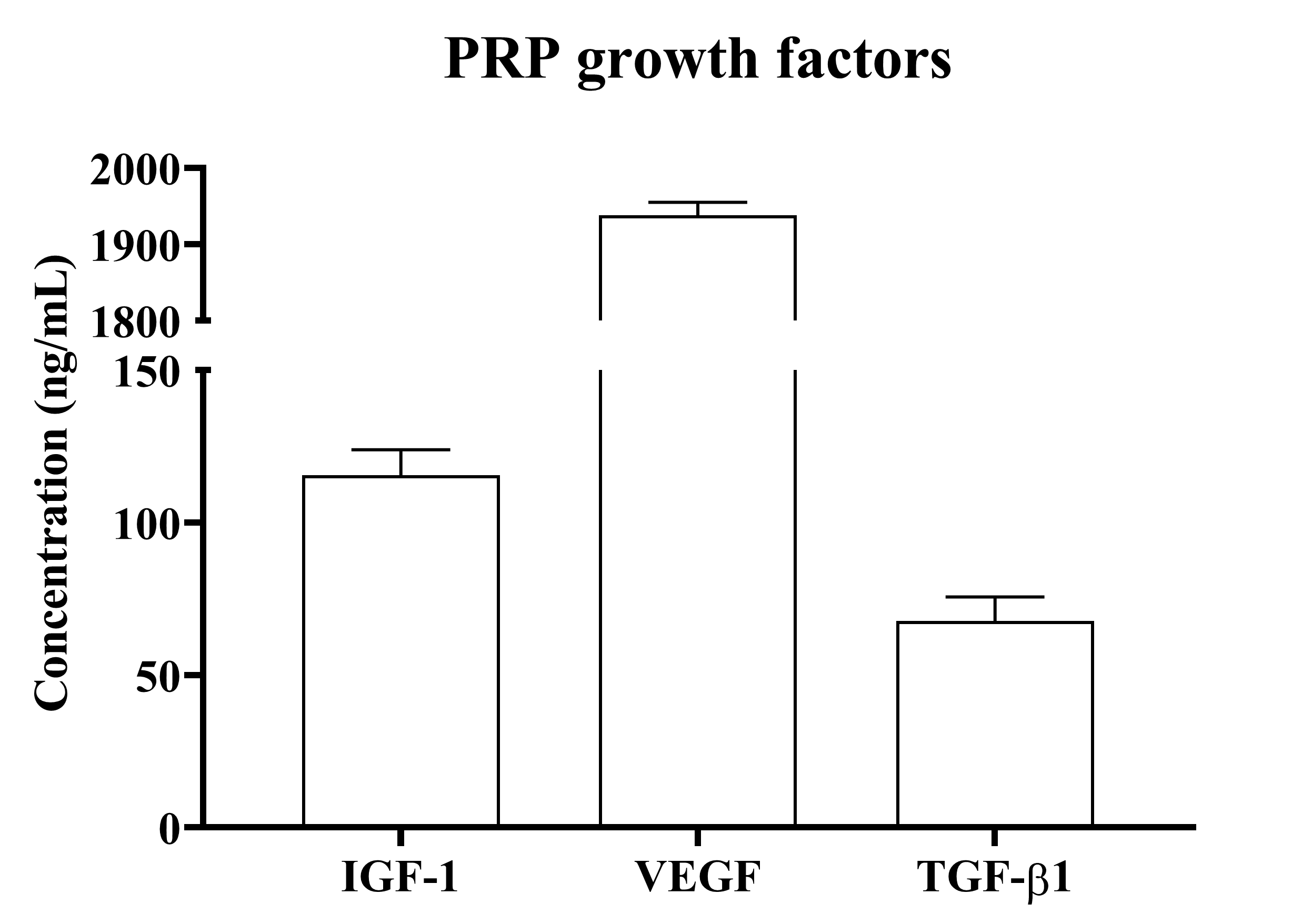

Supplement: Supplementary Figure 1 — Growth factors quantification in Platelet-rich plasma (PRP): insulin-like growth factor 1 (IGF-1; CSB-E13753Sh, Cusabio, Aurogene Srl, Rome, Italy), vascular endothelial growth factor A (VEGF-A; Quantikine ELISA kit, DVE00, R&D Systems, Bio-Techne SRL, Milan, Italy), and transforming growth factor beta 1 (TGF-β1; TGF-beta 1 DuoSet ELISA, DY240, R&D Systems, Bio-Techne SRL, Milan, Italy). All experiments were run following the protocol provided by the manufacturer. Data are expressed as mean ± SEM. [file Image_1.TIF]

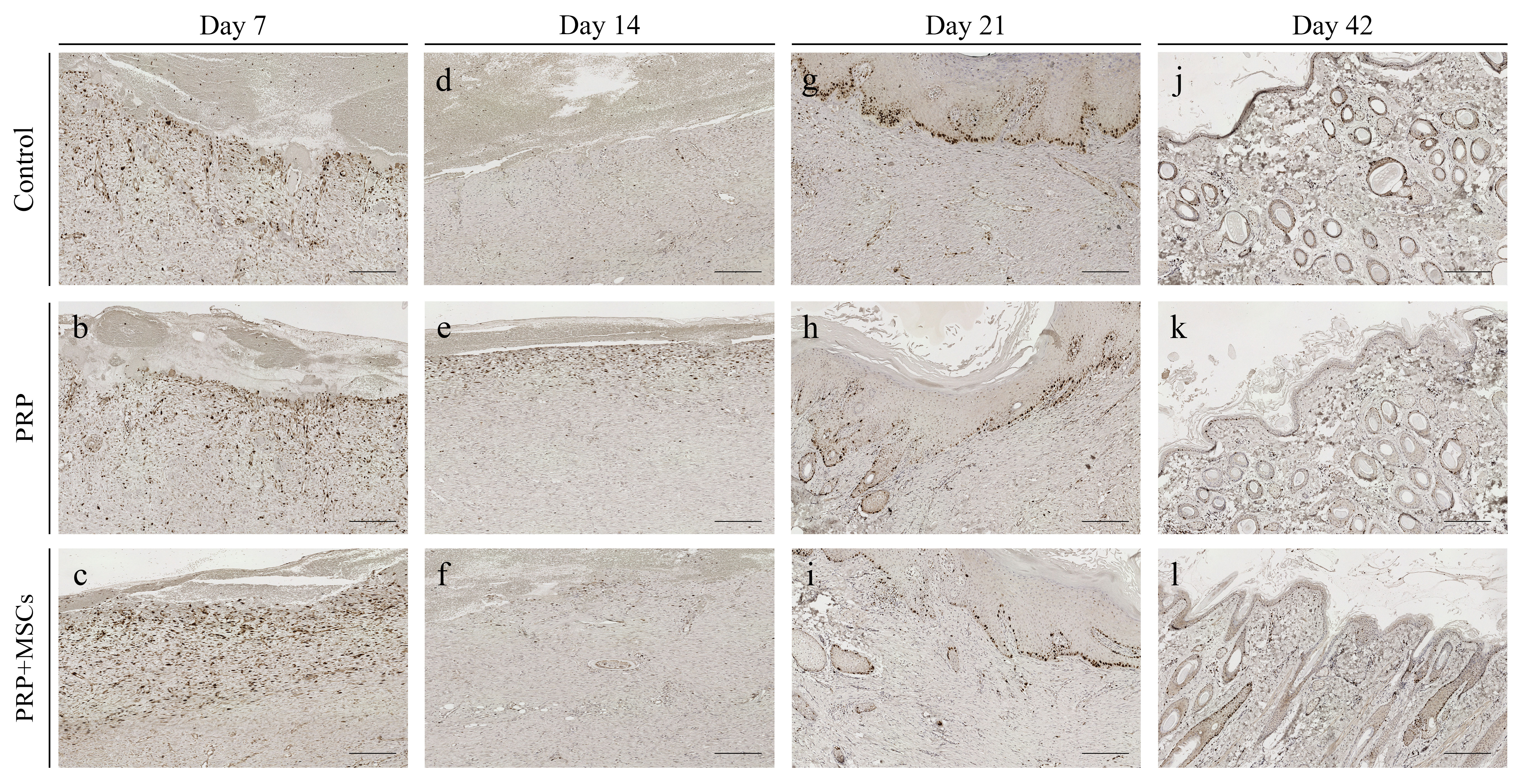

Supplement: Supplementary Figure 2 — Microphotographs for Ki67 immunolabeling. (A–C) Skin wounds at 7 days; (D–F) wounds at 14 days; (G–I) wounds at 21 days; (J–L) wounds at 42 days after wounding. Scalebar = 200 μm. [file Image_2.JPEG]

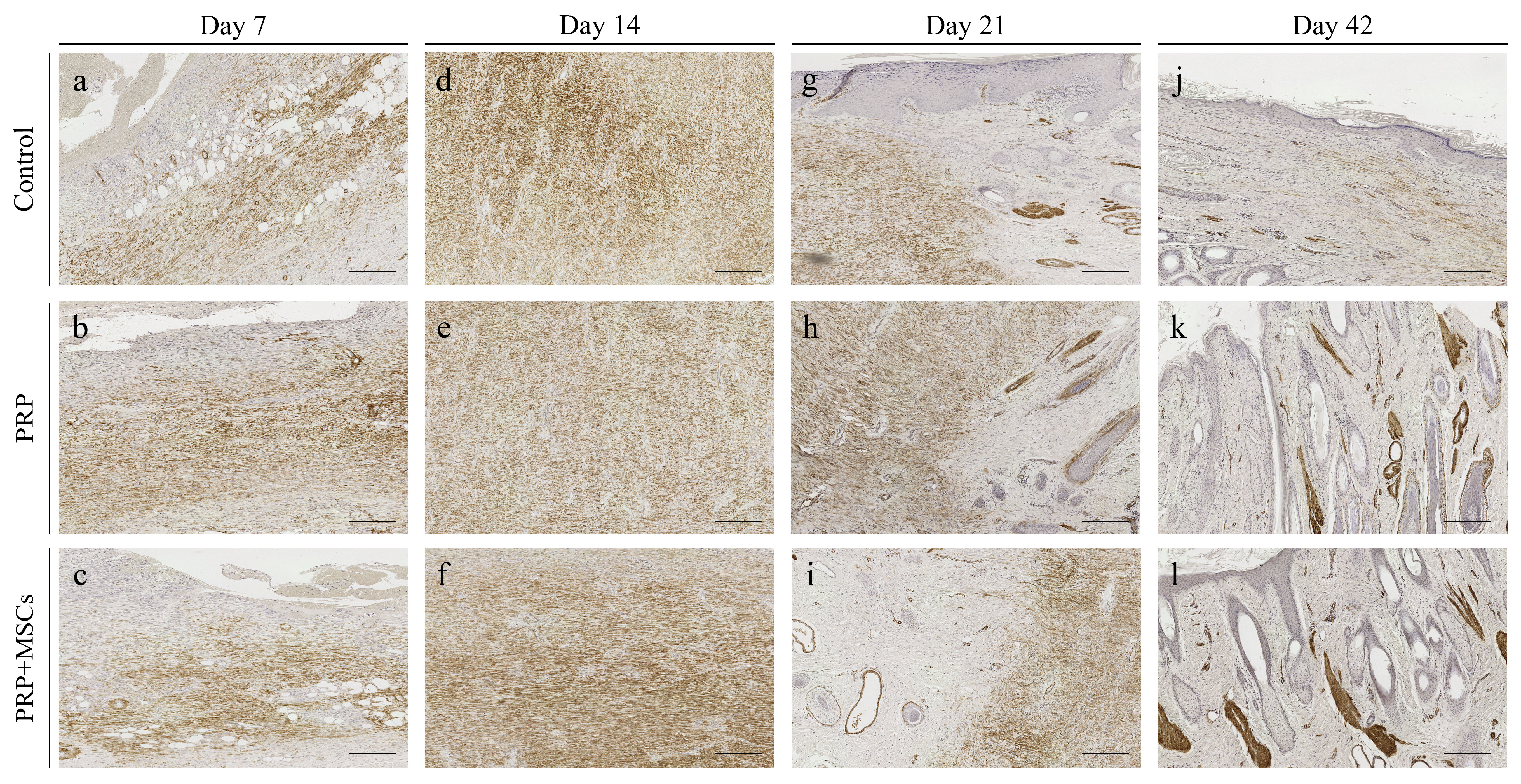

Supplement: Supplementary Figure 3 — Microphotographs for alpha smooth muscle actin (α-SMA) immunostaining. (A–C) Skin wounds at 7 days; (D–F) wounds at 14 days; (G–I) wounds at 21 days; (J–L) wounds at 42 days after wounding. Scalebar = 200 μm. [file Image_3.JPEG]
